# Biologically Active Compounds in *Stizolophus balsamita* Inflorescences: Isolation, Phytochemical Characterization and Effects on the Skin Biophysical Parameters

**DOI:** 10.3390/ijms22094428

**Published:** 2021-04-23

**Authors:** Joanna Nawrot, Jaromir Budzianowski, Gerard Nowak, Iwona Micek, Anna Budzianowska, Justyna Gornowicz-Porowska

**Affiliations:** 1 Department and Division of Practical Cosmetology and Skin Diseases Prophylaxis, Poznan University of Medicinal Sciences, Mazowiecka 33, 60-623 Poznan, Poland; joannac@ump.edu.pl (J.N.); gnowak.gerard@gmail.com (G.N.); micekiwonax@gmail.com (I.M.); 2 Department of Pharmaceutical Botany and Plant Biotechnology, Poznan University of Medical Sciences, SwMariiMagdaleny Str., 60-356 Poznan, Poland; jbudzian@ump.edu.pl (J.B.); abudzian@ump.edu.pl (A.B.)

**Keywords:** *Stizolophus balsamita*, flavonoids, simple phenolic compounds, sesquiterpene lactones, natural steroid

## Abstract

Three germacranolides, as well as five flavonoids, natural steroid and simple phenolic compounds, were isolated from the inflorescence of *Stizolophus balsamita* growing in Iran. The paper presents active compounds found for the first time in the inflorescence of this species. The flavonoids, simple phenolic compounds and natural steroids have been isolated for the first time in the genus *Stizolophus*. The MTT assay was employed to study in vitro cytotoxic effects of the taxifolin against human fibroblasts. We also evaluate the possible biological properties/cosmetic effects of *Stizolophus balsamita* extract and taxifolin on the human skin. Sixty healthy Caucasian adult females with no dermatological diseases were investigated. We evaluate the effects of *S. balsamita* extract and taxifolin on skin hydration and transepidermal water loss (TEWL). It was revealed that *S. balsamita* extract might decrease TEWL level and fixed the barrier function of the epidermis. The presence of bioactive phytochemical constituents in *S. balsamita* inflorescences makes them a valuable and safe source for creating new cosmetics and medicines.

## 1. Introduction

Medicinal plants are the potential source of compounds with a broad pharmacological spectrum of activity. Among the diverse functions, anti-inflammatory actions are highlighted [1]. The species belonging to the genus *Stizolophus*, subtribe Centaureinae (Asteraceae), are rich in compounds with chemotaxonomic significance—sesquiterpene lactones and flavonoids [2,3,4].

Sesquiterpene lactones have been isolated from all plant organs, but they most commonly occur in leaves and glandular trichomes on leaves. The wide variety of chemical structures is responsible for their distinctive biological activity, which is connected with the presence of α,β-unsaturated γ-lactone ring [5,6]. All sesquiterpene lactones, which have a lactone ring coupled with an exo-methylene, enables inhibition of the cellular enzymes through Michael nucleophilic addition [7]. In consequence, these compounds exhibit anti-inflammatory, antimigraine, antifungal, antibacterial and antiviral activity, including the SARS-CoV-2 virus [8,9]. Moreover, the additional substituent on C-8 and 4,5 epoxide group in germacranolides significantly increases the potency of the anti-inflammatory effect [8]. Sesquiterpene lactones also possess antiprotozoal [10], cytotoxic and antitumorigenic activity [5].

Flavonoids are natural compounds that are characterized by polyphenolic structures. They are abundantly found in all parts of the plants and are major contributors to the color and fragrance of fruits and flowers [11]. One of the more popular flavonoids isolated from the plants is quercetin and kaempferol from the group of flavones [12].

Flavonoids possess a wide range of health-promoting properties and are essential components in various pharmaceutical, cosmetics, nutraceutical and medicinal applications [13]. Flavonoids are known for their antioxidant activity to depend upon their molecular structure. The configuration, substitution, and a total number of hydroxyl groups substantially influence several antioxidant activity mechanisms such as radical scavenging and metal ion chelation ability [14]. The occurrence, position, structure, and total number of sugar moieties in flavonoids also play an important role in antioxidant activity. Aglycones are more potent antioxidants than their corresponding glycosides [15]. Moreover, it can be confirmed that the hydroxyl group at C-3 position is very important for the α-glucosidase inhibitory activity of flavonol compounds [16]. Flavonoids have been used extensively as anti-inflammatory [1], anticancer, antimicrobial, antiviral, antiangiogenic, antimalarial, neuroprotective, antitumor, and anti-proliferative agents [17]. The mechanism of anti-inflammatory activity is connected with the inhibition of several enzymes by modulating the arachidonic acid pathways. Thus, flavonoids have been the target of increasing interest as potential anti-inflammatory agents [1]. Flavonoids are also a promising group of compounds for treating neurodegenerative diseases like Alzheimer’s and Parkinson’s disease [18]. Furthermore, a broad range of skin benefits of flavonoids was postulated [19]. It was revealed that the aging effects caused by glycation of proteins degrades type I collagen differently and leads to an accelerated skin aging process [20]. In light of this, the positive effects on human skin and anti-glycation activity of taxifolin were previously suggested [19]. Therefore, novel, noninvasive cosmetological treatments and devices used in anti-aging strategies should be especially evaluated. For the assessment of skin biophysical parameters of skin aging, several tools have been developed and described in the research field of cosmetology and dermatology [21,22,23,24,25]. The instruments used in this study (Corneometer CM825, Tewameter TM300 from Courage-Khazaka Electronic, Köln, Germany) are considered to be noninvasive and therefore cause no harm or discomfort during the investigation of the skin parameters while accurately measure different aspects of the skin. Technical descriptions of this type of instrument and its use have been published by many authors [21,22,23,24,25].

Other groups of compounds which also occur in the subtribe Centaureinae are natural steroids and simple phenolic compounds [8].

Natural steroids are a class of chemical compounds (triterpenoids) synthesized by plants for defense against insects. Over 300 plant ecdysteroid analogs have been identified so far with distinctive chemical, physical and biological properties [26]. Phytoecdysteroids of the Centaureinae subtribe appear in the species of the *Serratula* genus [8]. Some pharmacological studies indicate the therapeutic benefits of phytoecdysteroids in humans. These compounds positively affect hypercholesterolemia, hyperglycemia, hypotension, infectious diseases (caused by viruses, bacteria, fungi), and physical and mental weakness [26]. The most highlighted pharmacological activity of phytoecdysones is their anti-inflammatory effect [27]. Nowadays, natural steroids are often chosen for skin change treatment, replacing synthetic steroids [8,26]. Natural steroids are also known for their antioxidant and anticancer activity [28,29].

Natural steroids (phytoecdysones) are found together with β-arbutin in species of the genus *Serratula* (Centaureinae). Our study has been shown that β-arbutin also accompanying phytoecdysones in the *Stizolophus* genus. This simple phenolic compound is practically used as a skin-lightening agent, and it has been reported to possess a weak antioxidant activity [30].

Another phenolic compound found during our study was an ester of protocatechuic acid (PCA). PCA is a type of widely distributed naturally occurring phenolic acid. It is present in rich quantity in various multiple fruits such as berries. PCA has been reported for its potential antioxidant activity, antibacterial activity, anticancer activity, antiulcer activity, antidiabetic activity, anti-aging activity, antifibrotic activity, antiviral activity, anti-inflammatory activity, analgesic activity, antiatherosclerotic activity, cardiac activity, hepatoprotective activity, neurological and nephroprotective activity [31].

The subject of our investigations was the inflorescences of *Stizolophus balsamita* (Lam.) K. Koch. (syn. *Stizolophus balsamitifolius* Cass.). It is an annual endemic plant that grows in Iran and Kazachstan. Its widely used in folk medicine in fever and externally in anginas and herpes [4]. The compounds that appear in *S. balsamitae* leaves were presented in our previous papers [1,2]. Now we decided to study, for the first time, the chemical content of *S. balsamita* inflorescences, and the results are described below.

Moreover, the present study’s additional aim was to investigate the influence of *Stizolophus balsamita* extract and its dominant compound taxifolin on the human skin. This interaction of examined compounds with skin was evaluated by noninvasive biophysical techniques using the Corneometer and Tewameter measuring the skin water content and skin barrier function of human volunteers, respectively. The potent cytotoxicity was determined on the human skin fibroblasts cell line.

## 2. Results

**Figure 1:**
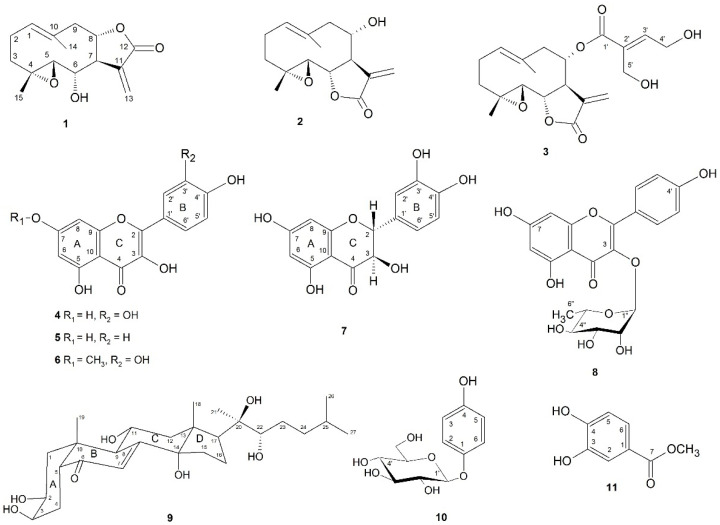
Chemical structures of compounds **1**–**11** (**1**—izospiciformin, **2**—stizolin, **3**—stizolicin, **4**—quercetin, **5**—kaempferol, **6**—rhamnetin, **7**—taxifolin (dihydroquercetin), **8**—kaempferol 3-*O*-α-rhamnopyranoside, **9**—ajugasterone C, **10**—arbutin, **11**—protocatechuic acid methyl ester).

Our phytochemical studies of the *S. balsamitae* inflorescentia led to the isolation and identification of three sesquiterpene lactones—germacranolides with 4,5-epoxide group—izospiciformin (**1**), stizolin (**2**) and stizolicin (**3**), as well as five flavonoids: quercetin (**4**), kaempferol (**5**), rhamnetin (**6**), taxifolin (**7**) and kaempferol 3-O-α-rhamnopyranoside (**8**), one natural steroid ajugasterone C (**9**), and two simple phenolic compounds: arbutin (**10**) and protocatechuic acid methyl ester (**11**) (Figure 1).

### 2.1. Isolation of Compounds from S. Balsamita Inflorescences

The inflorescences of *S. balsamita* (530 g) were air-dried at room temperature and finally crushed and soaked in 3.5L MeOH three times at room temperature. The MeOH extract was evaporated and the residue was dissolved in 0.5L H_2_O. The aqua solution was re-extracted three times with 0.25L AcOEt. The AcOEt extract was dried with anhydrous Na_2_SO_4_, filtrated and evaporated, giving a residue (12.45 g).

The AcOEt extract was chromatographed with CC method on silica gel with CH_2_Cl_2_, a mixture of CH_2_Cl_2_ andCH_3_OH (ratio 35:1) as eluent. The polarity was gradually increased with added CH_3_OH. Some collected fractions were subjected to repeated rechromatography until pure compounds were obtained. Fractions were rechromatographed on silica gel with CH_2_Cl_2_ and (CH_3_)_2_CO (ratio 10:1) or n-hexane and AcOEt (ratio 3:1). The polarity was gradually increased with added (CH_3_)_2_CO or AcOEt, respectively. The result was the isolation of the following compounds: izospiciformin (**1**) (3.4 mg, m.p. 192–195 °C), stizolin (**2**) (7.6 mg, m.p. 183–188 °C), stizolicin (**3**) (8.1 mg, m.p. 143–144 °C), quercetin (**4**) (1.8 mg, m.p 315–316 °C), kaempferol (**5**) (4.3 mg, m.p. 276–278 °C), rhamnetin (**6**) (0.9 mg, amorphous solid), taxifolin (**7**) (11.7 mg, m.p. 244–245 °C), kaempferol 3-O-rhamnoside (**8**) (2.1 mg, amorphous solid) and fraction 1—mixture of kaempferol 3-O-rhamnoside and ajugasterone C (**9**) (2.7 mg, amorphous solid) and fraction 2—mixtures with taxifolin, arbutin (**10**) and protocatechuic acid methyl ester (**11**) (2.3 mg, amorphous solid).

#### Spectral data of isolated compounds

Quercetin (**4**) ^1^H NMR (600 MHz, CD_3_OD): δ 7.59 (1H, d, *J* = 2.1, H-2), 7.51 (1H, dd, *J* = 8.5, 2.1 Hz, H-6), 6.86 (1H, d, *J* = 8.5 Hz, H-5), 6.20 (1H, d, *J* = 1.8 Hz, H-8), 6.04 (1H, d, *J* = 1.8 Hz, H-6).

Kaempferol (**5**) ^1^H NMR (600 MHz, CD_3_OD): δ 7.77 (2H, AA’, “d”, *J* = 8.8), 6.94 (2H, MM’, “d”, *J* = 8.8 Hz), 6.38 (1H, d, *J* = 2.1 Hz), 6.20 (1H, d, *J* = 2.1 Hz).

Rhamnetin (**6**) EI-MS (probe) m/z (relative abundance %): 316.0 [M]^+^ (100), 314.9 [M-H]^+^ (69), 302.0 [M-CH_3_]^+^ (19), 288 [M-CO]^+^ (37), 273.0 (37), 165.9 [A_1_]^+^ (21), 152.8 [M-CH_3_ A_1_]^+^ (63),136.8 [B_1_]^+^ (25).

Taxifolin (**7**) see Table 1.

Kaempferol 3-*O*-α-rhamnopyranoside (**8**) see Table 1.

Ajugasterone C (**9**) see Table 2.

Arbutin (**10**) ^1^H NMR (600 MHz, CD_3_OD): δ 6.96 (2H, AA’, ‘d’, *J* = 9.1 Hz, H-2,6), 6.69 (2H, MM’, ‘d’, *J* = 9.1 Hz, H-3,5), 4.73 (1H, d, *J* = 7.4 Hz, H-1 glc), 3.88 (1H, dd, *J* = 11.9, 1.7 Hz, H-6_A_, glc), 3.69 (1H, dd, *J* = 11.9, 5.6 Hz, H-6_B_, glc), 3.41 (1H, m, H-2 glc), 3.44-3.40 (m, H-3,4,5 glc). ^13^C NMR (150 MHz, CD_3_OD): δ 155.28 (C-1), 152.45 (C-4), 119.44 (C-2,6), 116.73 (C-3,5), 103.85 (C-1 glc), 78.62 (C-5), 62.36 (C-6).

Protocatechuic acid methyl ester (**11**) ^1^H NMR (600 MHz, CD_3_OD): δ 7.41 (1H, d, *J* = 2.0 Hz, H-2), 7.35 (1H, dd, *J* = 8.2, 2.0 Hz, H-6), 6.71 (1H, d, *J* = 8.2 Hz, H-5), 3.97 (3H, s, CH_3_O-7). ^13^C NMR (150 MHz, CD_3_OD): δ 172.02 (C-7), 152.00 (C-4), 145.28 (C-3), 123.12 (C-6), 117.89 (C-2), 115.43 (C-5), 52.75 (CH_3_O-7) (C-1 signal did not emerge from the background).

### 2.2. Identification of Isolated Compounds

The isolated compounds were identified by spectral methods.

Compounds **1**, **2** and **3** were identified as sesquiterpenes—izospiciformin, stizolin and stizolicin, respectively, by direct comparison with the samples isolated and identified from the leaves of the same plant (*S.balsamita*) [2].

Compound **4** showed an ABX system at δ_H_ 7.59 (d, *J* = 2.1), 7.51 (dd, *J* = 8.5, 2.1 Hz) and 6.86 (d, *J* = 8.5 Hz), and an AB system at δ_H_ 6.20 and 6.04 (each d, *J* = 1.8 Hz) in the ^1^H NMR spectrum (Figure S1) which were assigned to the flavonoid quercetin (3,5,7,3′,4′-tetrahydroxyflavone) [32] (Figure 1).

Compound **5** exhibited in the ^1^H NMR spectrum (experimental, Figure S2) an AA’BB’ system at 7.77 and 6.94 (each 2H, *J_ortho_ =* 8.8 Hz), and an AB system 6.38 and 6.20 (each 1H, d, *J* = 2.1 Hz) typical of the flavonoid kaempferol (3,5,7,4′-tetrahydroxyflavone) [33] (Figure 1).

Compound **6** was obtained in a minute amount, so only its mass spectrum was recorded. The EI-MS spectrum (Figure S3) showed a molecular ion [M]^+^ at *m/z* 316.0 consistent with a mass of methoxy–tetrahydroxy–flavone. The fragmentation ions corresponded to loss of a hydrogen and a methyl group at *m/z* 315 and 302, respectively. The fragment ions formed due to RDA (retro-Diels–Alder) degradation of the parent ion ([M]^+^) at *m/z* 165.9 (RDA-A_1_) and *m/z* 136.8 (RDA-B_2_) pointed to the ring A substituted with methoxy and hydroxy groups and the ring B bearing two hydroxy groups, respectively [34]. Thus, compound **6** was determined as rhamnetin, i.e., 7-methyl ether of quercetin (3,5,7,3′,4′-pentahydroxyflavone) [35] (Figure 1).

Compound **7** displayed a ^13^C NMR spectrum containing 15 carbon atoms signals including 12 signals of two aromatic rings in the shift range δ_C_168—95, two aliphatic oxymethine carbon signals at δ_C_85.17 and 73.73 and a low field carbonyl carbon at δ_C_198.41 (Table 1)(Supplementary Materials, Figure S5). Those data suggested a flavonoid of a dihydroflavonol type composed of the three rings A, B and C. The ^1^H NMR spectrum (Table 1, Figure S4), interpreted with the aid of an HH-COSY spectrum (Figure S6), showed an ABX system signals at δ_H_6.85 (dd, *J* = 7.8, 2.4 Hz), 6.80 (d, *J* = 7.8 Hz) and 6.96 (d, *J* = 2.4 Hz) ascribable to the protons H-6’, H-5’ and H-2’ of the ring B. The spectrum also showed an AB system of *meta*-coupled protons (*J* = 2.4 Hz) at δ_H_5.91 and 5.88 assigned to the ring A, and two vicinal aliphatic oxymethine protons of the ring C at δ_H_4.91 and 4.50. The two latter signals were each a doublet with a large coupling constant *J* = 11.8 Hz due to their *trans*-diaxial disposition. The NMR assignments of proton and carbon signals were verified by HSQC, HMBC and NOESY spectra (Table 1; Figures S7–S9). From those data, compound **7** was identified as 5,7,3′,4′-tetrahydroxydihydroflavonol (Figure 1) or dihydroquercetin, known by the trivial name—taxifolin [36].

The impure sample of compound **8** could be identified as kaempferol 3-*O*-α-rhamnopyranoside by ^1^H NMR only. The NMR data were identical to those found for the sample of **8** described below.

Fraction 1 contained two compounds—**8** and **9** according to NMR analyses (Table 1; Figures S10–S16). The ^1^H NMR spectrum of compound **8** (Table 1, Figure S10) exhibited an AA’XX’ system at δ_H_7.76 and 6.93, each with an *ortho* coupling *J* = 8.8 Hz, typical for the 1,4-disubstituted benzene ring, as well as an AB system of *meta*-coupled protons at δ_H_6.33 and 6.17 (each d, *J* = 2.1 Hz). Those signals were ascribed to the ring B H-2′,6’, H-3,’5’, and the ring A H-8, H-6, respectively, and responded to a flavonoid aglycone like kaempferol. The spectrum also contained signals of a sugar residue, which were traced with the HH-COSY spectrum starting from an anomeric proton signal at δ_H_5.37 to show a sequence of two equatorial and three axial oxymethine protons and a terminal methyl group (δ_H_0.92, d, *J* = 6.3 Hz)—all indicative of an α-rhamnopyranosyl. The HMBC spectrum coupling between the anomeric proton and a carbon signal at δ_H_136.10 allowed location of the glycosidic linkage at the C-3 of the aglycone. The α configuration of a glycosidic linkage was evidenced by a small coupling constant for the anomeric proton signal (*J* = 1.8 Hz) in the ^1^H NMR spectrum and a large magnitude of the coupling constant between the anomeric proton and carbon signals, *J* = 172 Hz, determined from the residual signals in the HMBC spectrum. Hence, compound **8** was identified as kaempferol 3-*O*-α-rhamnopyranoside (Figure 1) and the NMR spectra are in agreement with reported data [37,38].

Compound **9** exhibited the ^13^C NMR spectrum (Table 2, Figures S11–S12) with 27 carbon signals, including those corresponding to a carbonyl carbon at δ_C_206.63, an olefinic bond at δ_C_165.72 and 122.72, and carbon atoms substituted with five hydroxyl groups at δ_C_68.93, 68.56, 69.49, 77.75 and 77.95. Five methyl groups, as three singlets at δ_H_1.20, 1.05, 0.87, and two doublets at δ_H_0.92 and 0.91, were recognized in the ^1^H NMR spectrum (Table 2; Figure S10) interpreted with the aid of the HH-COSY spectrum (Figure S13). The olefinic proton (H-7) signal at δ_H_ 5.80(d) showed an allylic coupling (*J* = 2.7 Hz) with H-9 at δ_H_ 3.14 (dd). The latter exhibited a diaxial coupling (*J* = 8.9 Hz) with the low-field methine (H-11) signal at δ_H_ 4.10 due to the substitution with a hydroxyl group. The H-11 had a β-configuration from the NOESY spectrum (Figure S16) interactions with angular methyl signals at δ_H_ 0.87 (H-18) and 1.05 (H-19). The low-field, OH-substituted methine signal at δ_H_ 3.95 (H-3), exhibited HH-COSY couplings (*J* = 2.9 Hz) to methylene protons (H-4)at δ_H_1.77 (ddd) and 1.69 (ddd), which in turn showed HMBC couplings to the carbonyl carbon (C-6) signal at δ_C_ 206.63. The small vicinal couplings (*J* = 2.9 Hz) of H-3 signal indicated its equatorial orientation. H-3 was also coupled to an axial oxymethine at δ_H_4.00 (ddd) which showed geminal couplings with a methylene protons at δ_H_ 1.37(*J* = 4.3 Hz) and 2.58 (*J* = 11.9Hz) in the HH-COSY spectrum and hence were assigned to C-2 and C-1 positions, respectively. The H-5 proton signal at δ_H_2.33 (dd) had an axial orientation from diaxial (*J* = 13.1 Hz) and axial–equatorial (*J* = 4.0 Hz) couplings to H-4 protons. The spatial proximity between an axial H-1 (δ_H_1.37), H-5 and C-19 methyl protons, as well as between H-2 and H-9, revealed by interactions observed in the NOESY spectrum, required a *cis* fusion of A and B rings. Hence, compound **9** was identified as an ecdysteroid—ajugasterone C (Figure 1) and the obtained spectral data were comparable to those previously reported [39].

Fraction 2 appeared to contain a mixture of compounds **7** (taxifolin), **10** and **11** by the interpretation of its NMR spectra (experimental, Figures S17–S24). The ^1^H NMR signals of compound **10** involved signals an aromatic AA’XX’ system at δ_H_6.96 and 6.69 (each 2H, *J_ortho_* = 9.1 Hz) and signals ascribable to a β-linked glucopyranosyl due to the presence of an anomeric proton at δ_H_4.73 (d, *J* = 7.4 Hz, H-1’), oxymethines in the range 3.44-3.40 (H-2′-H-5’), and of an oxymethylene at δ_H_3.69 and 3.41 (H_2_-6’). Together with the carbon atom signals assigned by HSQC and HMBC spectra (Figures S23,S24), the NMR data of 10 fit well with those recorded in the same solvent (CD_3_OD) (unpublished) for an arbutin sample isolated previously from *Serratula quinquefolia* (Asteraceae) in our previous work [40] (Table S1, Figures S25–S28). Compound 11 showed an AMX system at δ_H_7.41 (d, *J* = 2.0 Hz), 7.50 (dd, *J* = 8.2, 2.0 Hz), 6.71 (d, *J* = 8.2 Hz), corresponding to an 1,3,4-trisubstituted benzene ring, and a singlet of a methoxyl at δ_H_3.97. The latter revealed an HMBC spectrum coupling to the carbon at δ_C_172.02 assigned to the carbonyl of the esterified carboxylic group. Therefore, compound 11 was identified as protocatechuic acid methyl ester (Figure 1), and the obtained NMR data fit well those reported for this compound [41].

### 2.3. The Biological Effect of S. Balsamita Extract on Human Skin

#### 2.3.1. In Vitro Cytotoxicity Experiments

**Figure 2:**
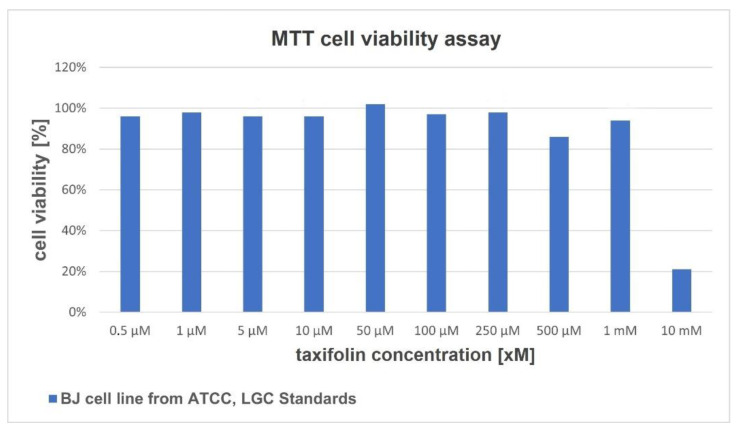
The MTT cell viability assay on a human skin fibroblast cell line. The results are expressed as cell viability in different taxifolin concentrations.

Cell viability deteremined by methylthiazolyldiphenyl-tetrazolium bromide (MTT) assay was 96% for 0.5 µM; 94% for 1mM and 21% for 10 mM concentration of taxifolin. The IC 50 value was calculated as > 6.24 nM. The obtained cell viability results for each taxifolin concentration are shown in Figure 2.

#### 2.3.2. Analysis of the effect of *S. balsamita* extract and taxifolin on human skin parameters

Detailed results of skin biophysical parameters (hydration and TEWL) comparison before and after treatment with examined creams are presented in Table 3.

At the baseline, there were no differences between groups (*p* > 0.05).

**Figure 3:**
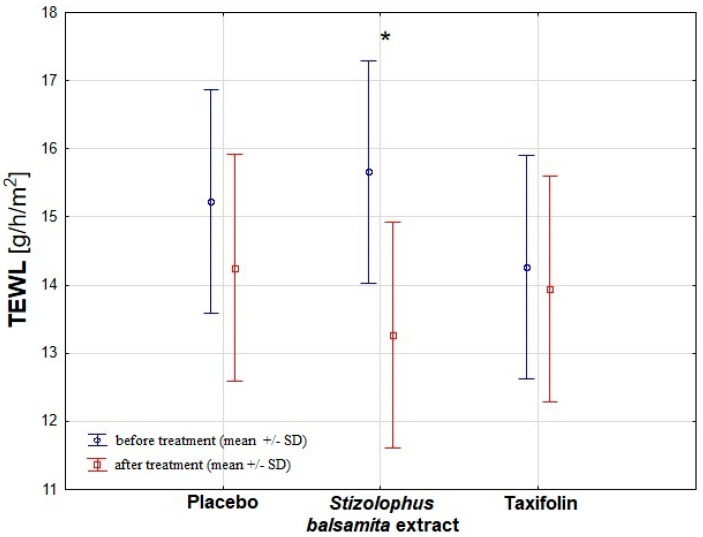
The comparison of skin TEWL results between examined groups. The results are expressed as the mean ± standard deviation (SD). Descriptions: *—statistically significant for *Stizolophus balsamita* extract before and after treatment.

The application of a cream with *Stizolophus balsamita* extract significantly improved skin barrier function compared with the taxifolin and the placebo control by decreasing the value of transepidermal water loss (TEWL) with statistical significance (*p* = 0.005; Figure 3).

**Figure 4:**
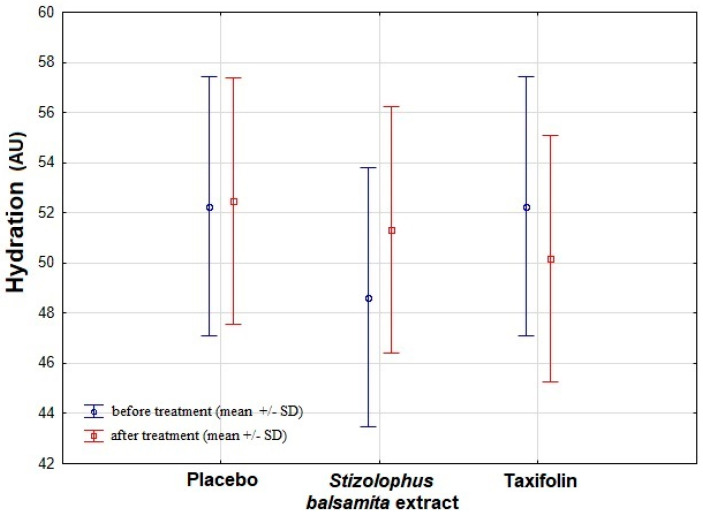
The comparison of skin hydration results between the examined groups. The results are expressed as mean ± standard deviation, SD (*p* < 0.05). There was no significant difference between skin hydration measured before and after treatment in examined groups. Descriptions: AU—arbitrary unit.

Skin hydration was enhanced on the face cheek treated with the *Stizolophus balsamita* extract in the examined group (Table 3). However, no statistically significant improvement in skin hydration was revealed (*p* > 0.05; Figure 4).

## 3. Discussion

The results obtained in this study indicate that *S.balsamita* inflorescences possess unique chemical composition—four groups of natural compounds: sesquiterpene lactones, flavonoids, natural steroid and simple phenolic compounds. Moreover, these compounds are characterised by a wide spectrum of pharmacological activity with a dominant anti-inflammatory effect [1,8,27,31].

Germacranolides **1**–**3** possess anti-inflammatory and antiserotonin effects. They inhibit the release of 5-HT from platelets more effectively than parthenolide, and the izospiciformin (**1**) shows the most potent effect [42]. It is worth noticing that all isolated germacranolides, along with the three elements characteristic for a parthenolide (4,5-epoxide, lactone ring and exo-methylene), have an additional substituent on C-8, which significantly increases the potency of the antiserotonin effect compared to parthenolide [42]. Stizolicin (**3**) also has remarkable cytotoxic and antiparasitic activities [3].

Flavonoids 4–8 were the major constituents of the *S.balsamita* inflorescences. Quercetin (**4**), one of the most common flavonoids in vegetables and fruits, was isolated with their derivatives rhamnetin (**6**) and taxifolin (**7**), which was yielded in a higher amount. Quercetin (**4**) primarily shows anti-inflammatory and antioxidant activity. It also possesses analgesic and inflammasome inhibitor activity [43]. Moreover, compound (**4**) has inhibitory activity against severe acute respiratory syndrome coronavirus (SARS-CoV) and Middle East respiratory syndrome coronavirus (MERS-CoV) [44]. Therefore, can be a potential treatment for severe inflammation, which is the main life-threatening condition in patients with COVID-19 [43]. The ability to inhibit coronavirus and its inflammatory processes is strongly desired in a new drug for the treatment of COVID-19 [45]. Furthermore, quercetin (**4**) has been used most effectively for colorectal cancer [46]. When present in the bloodstream, this antioxidant flavonoid improves vascular health and reduces the risk of cardiovascular disease in its conjugated form. Quercetin (**4**) and its derivatives prevent thrombosis or blood clotting and prevent chances of stroke [47].

Kaempferol (**5**) possesses anti-inflammatory effects. It has been shown to be a safe and efficacious natural dietary anti-inflammatory agent in both in vivo and in vitro studies [12]. It was also found that kaempferol is a potential anti-atherogenic agent which prevents vascular inflammation [48] and possesses antidiabetic [49], antiviral [17] activity. Kaempferol also can reduce the risk of cancer. It stimulates the body’s antioxidants against free radicals that cause cancer [50].

Rhamnetin (**6**) is a methylated derivative of quercetin. The presence of 3OH on the C-ring of rhamnetin may contribute to both its anti-inflammatory and enzymatic inhibition of secretory phospholipase A2 (sPLA2), and the methylation of ring A may provide the increase in cell viability and a low creatine kinase (CK) level induced by sPLA2 [51]. These results showed that compound (**6**) could be a candidate as a natural compound for the development of new anti-inflammatory drugs. Both rhamnetin (**6**), as well as quercetin (**4**) exert strong antioxidant activity. These properties could allow administering flavonoids for the prevention of numerous free-radical-based diseases or as an additive element to the food and pharmaceutical industry [52].

Taxifolin (**7**) is a common flavanonol that exerts various pharmacological activities, including antioxidant, anti-inflammatory, antiviral, antibacterial activities, anticancer, hepatoprotective and neuroprotective activities [53,54,55]. Recently, taxifolin has been reported to inhibit osteoclastogenesis, so it may be considered as a potential alternative therapeutic agent for treating osteoclast-related diseases [56].

Kaempferol 3-*O*-rhamnoside (**8**) has been shown to inhibit the proliferation of breast cancer cells and the absorption of dietary glucose in the intestines [57,58]. Moreover, this compound was shown to protect against beta-amyloid-induced cell death by inhibiting the self-assembly of beta amyloids [59]. Therefore, kaempferol 3-*O*-rhamnoside could serve as a potential treatment of cancer and Alzheimer’s disease.

The steroid character of ajugasterone C (**9**) decides its potent anti-inflammatory activity and strengthening effect (increases immunity, muscle mass, and erythropoiesis) [60]. The immunomodulatory activity of ecdysones concerns synthetic steroids and phytoecdysones. The presence of OH groups in the steroid skeleton of phytoecdysones makes them act without side effects, usually observed during the use of synthetic steroids [60,61,62]. Compound 9 can be used in preparation for treating skin changes caused by seborrheic dermatitis and as anti-*Malassesia restricta* agent [8,63].

β-arbutin (**10**) is a simple phenol glucoside known for its antibacterial activity. Therefore, arbutin-containing plant substances are used mostly in the treatment of urinary tract infections [64]. β-arbutin (**10**) also possesses an anti-inflammatory effect. Its mechanism of action is based on inhibiting the activity of tyrosinase, a vital enzyme in the process of melanin synthesis [65]. Compound (**10**) in cream is used as a first choice in treating hyperpigmentation [66]. The lack of significant adverse effects of arbutin and its derivatives makes them a valuable alternative to hydroquinone. Therefore, an increasing interest in arbutin and its derivatives is observed especially in the cosmetics industry [64].

Kakkar et al. [31] demonstrated that protocatechuic acid methyl ester (**11**) possess significant neuroprotective activities against glutamate-induced neurotoxicity.

It is known that retention of water in the *stratum corneum* plays an important role in the regulation of skin function. Loss of water may disturb skin appearance leading to various skin disorders. We assessed here the possible extent of damage to the epidermal barrier by determining TEWL, which is a commonly accepted sensitive indicator of disruptions in the epidermal lipid barrier. We revealed that *S. balsamita* extract might decrease TEWL level and, in this way, fixed the barrier function of the epidermis. It should be noted that there is a lack of significantly decreased TEWL in both control groups (taxifolin and placebo). Thus this biological property is specific for *S. balsamita* extract.

The literature shows that isolated compounds from *S. balsamita* inflorescences make it a promising source for the pharmaceutical and cosmetics industry.

## 4. Materials and Methods

### 4.1. General

The compounds were separated by column chromatography (CC) on silica gel (particle size: 0.063–0.200 mm; Merck, Darmstadt, Germany, Art. 7734). Selected fractions were further rechromatographed on silica gel with particle sizes of <0.063 mm (Merck, Darmstadt, Germany Art. 7729). The NMR spectra were run on a Bruker Avance 600 (Billerica, MA, USA) instrument using 600 and 150 MHz frequencies for hydrogen nuclei (^1^H) and carbon nuclei (^13^C), respectively, and tetramethylsilane (TMS) was used as an internal standard. The spectra were obtained for CDCl_3_ or DMSO-d_6_ solutions at 298 K. Chemical shifts are given in ppm, and coupling constants *J* are given in Hz. Melting points were determined on a Büchi B-540 (Essen, Germany) apparatus and are uncorrected. EI-MS spectra were recorded on an AMD Intectra Mass AMD 402 spectrometer (Harpstedt, Germany).

Preliminary phytochemical analysis of *S. balsamita* extract was carried out by thin layer chromatography method. TLC was performed on aluminium-backed silica gel plates (Merck, Darmstadt, Germany, Art. 5533). The plates were viewed under UV light (254 nm) or sprayed with anisaldehyde–sulfuric acid reagent (anisaldehyde 0.5 mL, glacial acetic acid 10 mL, methanol 85 mL, concd. sulphuric acid 4.5 mL) and heated at 103 °C for 3-4 min.

The study on human participants was approved by a local bioethical committee (Poznan University of Medical Sciences, no. 356/19, obtained 07 March 2019, Poland). Written informed consent was obtained from all participants.

### 4.2. Plant Material

Inflorescences of *Stizolophus balsamita* (Lam.) K. Koch (Asteraceae) were collected from the Botanical Garden of the Department and Division of Practical Cosmetology and Skin Diseases Prophylaxis, University of Medical Sciences in Poznan (Poland), where the voucher specimens (voucher numbers: 55/2014) are deposited. Seeds of *S. balsamita* were provided by the Botanical Garden in Teheran (Iran), which were gathered from the natural habitat in Iran. The plant was identified by our botanist based on the information from Flora Iranica [67] and Flora Europea [68].

### 4.3. In Vitro Cytotoxicity Experiments

An MTT assay was employed to measure the cytotoxic effects of taxifolin on human dermal fibroblast (the BJ cell line from ATCC, LGC Standards; ATCC^®^ CRL-2522™). Human dermal fibroblasts were cultured in EMEM medium supplemented with 5% fetal bovine serum, 4 mM L-glutamine and 1% penicillin–streptomycin. Briefly, cells were seeded into flat-bottomed 96-well cell culture plates. The cells were then treated with varying concentrations of taxifolin (Cyherb, Ciyuan Biotech, Xi’an, China). The dimethyl sulfoxide (DMSO) was used as a solvent. Positive (1% SDS) and negative (incubation only in culture medium) controls were conducted. The stock of ten taxifolin dilutions was prepared (0.5 µM, 1 µM, 5 µM, 10 µM, 50 µM, 100 µM, 250 µM, 500 µM, 1mM, 10mM) and each concentration of 100 µl was added in six repetitions to the respective wells. The plate was incubated at 37 °C in a humidified 5% CO_2_ incubator. Non-treated control cells were also maintained for comparing growth inhibition. The entire plate was observed after 24 h of treatment in a contrast tissue culture microscope in order to find any detectable variations in the morphology of the cells.

The sample content in the wells was removed after 24 h of the incubation period and was rinsed with phosphate-buffered saline (PBS) with calcium and magnesium ions. Subsequently, 100 µl of reconstituted MTT solution (0.5 mg/mL) was placed in all test and control wells. The plate was incubated for 3 h at 37 °C in a CO_2_ incubator.

The MTT was removed and 100 µl of isopropanol was added. Then, the wells were mixed for 15 min in order to solubilize the insoluble formazan crystals.

The absorbance values were measured at a wavelength of 570 nm with a microplate reader (TECAN Spark 10M, Männedorf, Switzerland). The IC50 value was calculated.

### 4.4. Examined Groups and Cream Preparation for Skin Measurements

The examined groups included a total of 60 individuals: (i) 20 subjects used a cream with *Stizolophus balsamita* extract (3%); (ii) 20 subjects used a cream with taxifolin (3%); (iii) 20 subject used a placebo (only cream base). All of them were in a good general state of health.

The recipe for the cream was developed by the Department and Division of Practical Cosmetology and Skin Diseases Prophylaxis, Poznan University of Medical Sciences (Poznan, Poland). The active substance of the cream was a dry ethanol extract of the *Stizolophus balsamita* containing taxifolin as a dominant compound and commercially available taxifolin obtained from Cyherb (Ciyuan Biotech, Xi’an, China). In this study, the examined ointments were prepared on the basis of a creamy, commercially available, multi-component medium with a pH close to the skin condition Lekobaza^®^ Pharma Cosmetic base (Fagron, Kraków, Poland).

Prior to the measurements, the volunteers were asked to stay in the test room for at least 15 min before the measurements, so the skin could acclimatize to room conditions. In order to minimize the errors during research, the volunteers were asked not to apply any cosmetic cream in the tested area before the study. Additionally, solar exposure was forbidden.

### 4.5. The Measurement of Biophysical Skin Parameters

The effect of the taxifolin and *S. balsamita* extract on skin biophysical parameters was performed according to the guidelines for the assessment of skin properties in non-clinical settings [21,22].

The range of hydration and skin barrier function measurements was performed on adult healthy female volunteers (*n* = 60) of different ages varying from 35 to 61 years (mean age 44.15).

The skin properties were measured with the use of noninvasive skin bioengineering techniques. All techniques for the skin and biophysical parameters evaluation were used with the Courage-Khazaka MPA-9 device with probes: Corneometer CM825, Tewameter TM300, (Courage-Khazaka Electronic, Köln, Germany) to evaluate the effects of examined creams on skin hydration and transepidermal water loss (TEWL).

The Corneometer^®^ operates at a low frequency (40–75 Hz) and measures the electrical capacitance of the *stratum corneum*. Since water has the highest di-electrical constant in the skin, capacitance values will increase with an increase in water content/skin hydration. The mean of three measurements are displayed in arbitrary units ranging from 0 to 130. TEWL was assessed by evaporimeter.

Skin water content (Corneometer CM 825, Courage Khazaka, Germany) and skin barrier function (Tewameter TM 300, Courage Khazaka, Germany) were measured before application and after 30 days of treatment (twice a day). Each patient used 3 mg of the cream.

The measurements were carried out on an exactly designated skin region (left cheek—2 cm below orbitale on the left side of the face in interpupillary line) for the repeatability measurements. For each chosen skin site, a test area (3 × 3 cm) was delimitated. The probe was applied to the skin surface (1.54 cm^2^). Three individual measurements were carried out for each cream at any time point using the Corneometer and Tewameter and the average value was used to calculate the results.

All measurements were performed in controlled conditions at a temperature of 22–25 °C and with an average relative humidity of 52–58%.

### 4.6. Statistical Analysis

Statistical analysis was based on Software Statistica PL 10.0 (StatSoft, Inc, Tulsa, OK, USA). All results were first verified by a normality test (Shapiro–Wilk test) which confirmed the compliance with the Gaussian curve. The repeated measure ANOVA test was performed to compare all results between groups and to compare results before and after treatment in three examined groups. When differences were found Bonferroni’s test was used. The assumed statistical significance was *p* < 0.05.

## 5. Conclusions

Results of this study and earlier studies point out that *Stizolophus balsamita* is rich in compounds with potent biological activity.

Germacranolides isolated from the *Stizolophus balsamitae* leaves exhibit antiviral activity, as well as an anit-inflammatory and antimigraine effect.

*S. balsamita* inflorescences rich in flavonoids: quercetin (**4**), kaempferol (**5**), rhamnetin (**6**), taxifolin (**7**) with antioxidant and anti-inflammatory activity, is an excellent material to prepare valuable pharmaceutical and cosmetic products. Taxifolin, a dominant compound in studied extract (on the base TLC analysis) is a commercial product on the pharmaceutical market.

We revealed that *S. balsamita* extract might decrease TEWL level and, in this way, fixed the barrier function of the epidermis, which is its specific biological property. We confirmed the bio-safe nature of taxifolin in relation to human fibroblasts.

## Figures and Tables

**Table 1:** NMR Data of Compounds **7** and **8**.

	Compound 7 (Taxifolin)		Compound 8 (Kaempferol 3-*O-*α-Rhamnopyranoside; Afzelin)	
Carbon	^13^C δ_C_	^1^H δ_H_ (Hz)	^13^C δ_C_	^1^H δ_H_ (Hz)
2	85.17	4.91 d (11.8)	158.70	-
3	73.73	4.49 d (11.8)	136.10	-
4	198.42	-	179.43	-
5	165.42	-	159.04	-
6	97.41	5.92 d (2.4)	100.51	6.17 d (2.1)
7	168.93	-	165.72	-
8	95.36	5.87 d (2.4)	95.23	6.33 d (2.1)
9	164.56	-	158.30	-
10	101.86	-	103.52	-
1’	129.97	-	122.72	-
2′	115.43	6.96 d (2.4)	131.87	7.76 d (8.8)
3′	146.38	-	116.56	6.93 d (8.8)
4′	147.20	-	161.65	-
5′	116.14	6.80 d (7.8)	116.56	6.93 d (8.8)
6′	120.90	6.84 dd (2.4, 7.8)	131.87	7.76 d (8.8)
1′’	-	-	103.57	5.37 d (1.8) (172) **
2′’	-	-	72.02	4.22 dd (1.8, 3.4)
3′’	-	-	71.94	3.70 dd (3.4, 9.1)
4′’	-	-	73.22	3.32 m *
5”	-	-	72.15	3.33 m *
6”	-	-	17.65	0.92 d (6.3)

* signal pattern unclear due to the overlap.** ^1^*J*_HC_ determined from the HMBC spectrum.

**Table 2:** NMR Data of Compound **9**.

	Compound (9) (Ajugasterone C)	
Carbon	^13^C δ_C_	^1^H δ_H_ (Hz)
1	39.08	2.58 dd (12.8, 4.3); 1.37 dd (12.8, 11.9)
2	68.93	4.00 ddd (11.9, 4.3, 2.9)
3	68.56	3.95 ddd (2.9, 2.9, 2.9)
4	33.29	1.77 ddd (14.0, 13.1, 2.9); 1.69 ddd (14.0, 4.0, 2.9)
5	52.77	2.33 dd (13.1, 4.0)
6	206.63	-
7	122.72	5.80 d (2.7)
8	165.72	-
9	42.93	3.14 dd (8.9, 2.7)
10	39.90	-
11	69.49	4.10 ddd (10.5, 9.0, 6.1)
12	43.78	2.20 dd (12.1, 10.5); 2.16 dd (12.1, 6.1)
13	49.08	-
14	84.86	-
15	31.83	1.97 m; 1.57 m
16	21.52	2.00 m; 1.71 m
17	50.28	2.41 dd (9.5, 8.6)
18	18.87	0.87 s (3H)
19	24.61	1.05 s (3H)
20	77.75	-
21	20.96	1.20 s (3H)
22	77.95	3.30 m
23	30.48	1.56 m; 1.22 m
24	37.65	1,46 m; 1.23 m
25	29.22	1.57 m
26	23.41	0.92 d (6.1) (3H)
27	22.74	0.91 d (6.1) (3H)

**Table 3:** Detailed results of skin biophysical parameters comparison before and after treatment with the *Stizolophus balsamita* extract and taxifolin.

Parameter	Examined Cream	Before Treatment	After Treatment	Results of Repeated Measure ANOVA (*p*)		
		Mean ± SD	Mean ± SD	Group	Time	Group * Time
TEWL [g/h/m^2^]	Extract of *Stizolophus balsamita* (3%) (*n* = 20)	15.66 ± 2.73	13.27 ± 4.03	0.83	0.005 *	0.132
	Taxifolin (3%) (*n* = 20)	14.27 ± 4.15	13.94 ± 3.82			
	Placebo (*n* = 20)	15.23 ± 3.93	14.26 ± 3.24			
Hydration (AU)	Extract of *Stizolophus balsamita* (3%) (*n* = 20)	48.62 ± 11.44	51.33 ± 12.93	0.778	0.769	0.138
	Taxifolin (3%) (*n* = 20)	52.25 ± 12.02	50.18 ± 9.35			
	Placebo (*n* = 20)	52.25 ± 11.15	52.46 ± 10.33			

Descriptions: *n*—number of volunteers; SD—standard deviation; AU—arbitrary unit; *—statistically significant for *Stizolophus balsamita* extract before and after.

## Data Availability

The data that support the findings of this study are available from the corresponding author upon reasonable request.

## Supplementary Materials

Supplementary materials can be found at https://www.mdpi.com/article/10.3390/ijms22094428/s1. spectra of compounds **4–11**, NMR data of the reference compound **10**.

## References

[1] Nunes C., Arantes M.B., Pereira S.M., Cruz L.L., Passos M., Moraes L.P., Vieira I.J.C., Oliveira D.B. (2020). Plants as sources of anti-inflammatory agents. Molecules.

[2] Nawrot J., Budzianowski J., Nowak G. (2019). Phytochemical profiles of the leaves of *Stizolophusbalsamita* and *Psephellussibiricus* and their chemotaxonomic implications. Phytochemistry.

[3] Suleimenov E.M., Morozowa O.V., Raidugin V.A., Gatilov Y.V., Rybalova T.V., Shakirov M.M., Seidakhmentov R., Aksartov R.M., Adekenov S.M. (2005). Sesquiterpene lactones from Stizolophusbalsamita and their biological activity. Chem. Nat. Comp..

[4] Suleimenov E.M., Raldugin V.A., Adekenov S.M. (2008). Cirsimaritin from Stizolophusbalsamita. Chem. Nat. Comp..

[5] Picman A.K. (1996). Biological activities of sesquiterpene lactones. Biochem. Syst. Ecol..

[6] Matejić J., Šarac Z., Randelović V. (2010). Pharmacological activity of sesquiterpene lactones. Biotechnol. Biotechnol. Equip..

[7] Heptinstall S., Groenwegen W.A., Spangenberg P., Loesche W. (1987). Extracts Feverfew may inhibit behavior via naturalization of sulphydryl groups. J. Pharm. Pharm..

[8] Nawrot J., Gornowicz-Porowska J., Nowak G. (2020). Phytotherapy perspectives for Treating Fungal Infections, Migraine, Sebhorreic Dermatitis and Hyperpigmentations with the Plants of the CentaureinaeSubtribe (Asteraceae). Molecules.

[9] Wyganowska-Swiatkowska M., Nohawica M., Grocholewicz K., Nowak G. (2020). Influence of Herbal Medicines on HMGB1 Release, SARS-CoV-2 Viral Attachment, Acute Respiratory Failure, and Sepsis. A Literature Review. Int. J. Mol. Sci..

[10] Hadas E., Derda M., Nawrot J., Nowak G., Thiem B. (2017). Evaluation of the amoebicidal activities of *Centaureabella*, *Centhaurheadaghestanica*, *Rhaponticumpulchrum* and *Tanacetumvulgarae* against pathogenic *Acanthamoeba* spp. Acta Pol. Pharm..

[11] Havsteen B. (2002). The biochemistry and medical significance of the flavonoids. Pharmacol. Ther..

[12] Alam W., Khan H., Shah M.A., Caul O., Saso L. (2020). Kaempferol as a dietary anti-inflammatory agent: Current therapeutic standing. Molecules.

[13] Panche A., Diwan A.D., Chandra S.R. (2016). Flavonoids: An overview. J. Nutr. Sci..

[14] Kelly E.H., Anthony R.T., Dennis J.B. (2002). Flavonoids antioxidants: Chemistry, metabolism and structure-activity relationships. J. Nutr. Biochem..

[15] Kumar S., Pandey A.K. (2013). Chemistry and biological activities of flavonoids: An overview. Sci. World J..

[16] Utari F., Itam A., Syafrizayanti S., Putri W.H., Ninomiya M., Koketsu M., Tanaka K., Efdi M. (2019). Isolation of flavonolrhamnosides from *Pometiapinnata* leaves and investigation of α-glucosidase inhibitory activity of flavonol derivatives. J. Appl. Pharm. Sci..

[17] Ullah A., Munir S., Badshah S.L., Khan N., Ghani L., Poulson B.G., Emwas A.-H., Jaremko M. (2020). Important Flavonoids and their role as therapeutic agent. Molecules.

[18] Teles R.B., Diniz T.C., Pinto T.C.C., Júnior R.G., Silva M.G., Lavor E.M., Fernandes A.W.C., Oliveira A.P., Ribeiro F.P.R., Silva A.A.M. (2018). Flavonoids as Therapeutic Agents in Alzheimer’s and Parkinson’s Diseases: A Systematic Review of Preclinical Evidences. Oxidative Med. Cell. Longev..

[19] Muramatsu D., Uchiyama H., Kida H., Iwai A. (2019). Cell cytotoxity and anti-glycation activity of taxifolin-rich extract from Japanese larch, Larix kaempferi. Heliyon.

[20] Addor F.A.S. (2018). Beyond photoaging: Additional factors involved in the process of skin aging. Clin. Cosmet. Investig. Dermatol..

[21] du Plessis J., Stefaniak A., Eloff F., John S., Agner T., Chou T.C., Nixon R., Steiner M., Franken A., Kudla I. (2013). International guidelines for the in vivo assessment of skin properties in non-clinical settings: Part 2. transepidermal water loss and skin hydration. Skin Res. Technol..

[22] Berardesca E., Loden M., Serup J., Masson P., Rodrigues L.M. (2018). The revised EEMCO guidance for the in vivo measurement of water in the skin. Skin Res. Technol..

[23] Darlenski R., Sassning S., Tsankov N., Fluhr J.W. (2009). Noninvasive in vivo methods for investigation of the skin barrier physical properties. Eur. J. Pharm. Biopharm..

[24] Fox L.T., du Plessis J., Gerber M., van Zyl S., Boneschans B., Hamman J.H. (2014). In Vivo skin hydration and anti-erythema effects of Aloe vera, Aloe ferox and Aloe marlothii gel materials after single and multiple applications. Pharmacogn. Mag..

[25] Enright K.M., Nikolis A. (2020). In vivo determination of the skin surface topography and biophysical properties of human hands: Effects of sex and hand dominance. Skin Res. Technol..

[26] Dinan L. (2001). Phytoecdysteroids: Biological aspects. Phytochemistry.

[27] Ochieng C.O., Ishola I.O., Opiyo S.A., Manguro L.A., Owuor P.O., Wong K.C. (2013). Phytoecdysteroids from the stem bark of *Vitexdoniana* and their anti-inflammatory effects. Planta Med..

[28] Nsimba R.Y., Kikuzaki H., Konishi Y. (2008). Ecdysteroids act as inhibitors of calfskin collagenase and oxidative stress. J. Biochem. Mol. Toxicol..

[29] Chen H., Tang B.Q., Chen L., Liang J.Y., Sun J.B. (2018). Neo-clerodane diterpenes and phytoecdysteroids from *Ajugadecumbens*Thunb. and evaluation of their effects on cytotoxic, superoxide anion generation and elastase release in vitro. Fitoterapia.

[30] Takebayashi J., Ishii R., Chen J., Matsumoto T., Ishimi Y., Tai A. (2010). Reassessment of antioxidant activity of arbutin: Multifaceted evaluation using five antioxidant assay systems. Free Radic Res..

[31] Kakkar S., Bais S. (2014). A review on Protocatechuic Acid and Its Pharmacological Potential. ISRN Pharmacol..

[32] Lu H., Yang S.H., Ma H., Han Z.H., Zhang Y. (2016). Bioassay-guided separation and identification of anticancer compounds in Tageteserecta, L. flowers. Anal. Methods.

[33] Lee S.B., Chung D. (2014). Synthesis and purification of kaempferol by enzymatic hydrolysis of tea seed extract. Biotechnol. Bioprocess Eng..

[34] Mabry T.J., Markham K.R., Harborne J., Mabry T.J., Mabry H. (1975). Mass spectrometry of flavonoids. The Flavonoids.

[35] Guzmán-Gutiérrez S.L., Nieto-Camacho A., Castillo-Arellano J.I., Huerta-Salazar E., Hernández-Pasteur G., Silva-Miranda M., Argüello-Nájera O., Sepúlveda-Roble S.O., Espitia C.I., Reyes-Chilpa R. (2018). Mexican Propolis: A source of antioxidants and anti-inflammatory compounds, and isolation of a novel chalcone and ε-caprolactone derivative. Molecules.

[36] Joo S.-J., Park H.-J., Park J.-H., Cho J.-G., Kang J.-H., Jeong T.-S., Kang H.C.H., Lee D.-Y., Kim H.-S., Byun S.-Y. (2014). Flavonoids from *Machilus japonica* Stems and their inhibitory effects on LDL oxidation. Int. J. Mol. Sci..

[37] Suedee A., Tewtrakul S., Panichayupakaranant P.H. (2013). Anti-HIV-1 integrase compound from *Pometia pinnata* leaves. Pharm. Biol..

[38] Tram N.C.T., Son N.T., Thao D.T., Cuong N.M. (2016). Kaempferol and kaempferol glycosides from *Phyllanthusacidus*leaves. Vietnam J. Chem..

[39] Budesínský M., Vokác K., Harmatha J., Cvacka J. (2008). Additional minor ecdysteroid components of *Leuzeacarthamoides*. Steroids.

[40] Nycz J.E., Malecki G., Morąg M., Nowak G., Ponikiewski L., Kusz J., Switlicka A. (2010). Arbutin: Isolation, X-ray structure and computational studies. J. Mol. Struct..

[41] Lee S.S., Kim T.H., Lee E.M., Lee M.H., Lee H.Y., Chung B.Y. (2014). Degradation of cyanidin-3-rutinoside and formation of protocatechuic acid methyl ester in methanol solution by gamma irradiation. Food Chem..

[42] Nawrot J., Napierała M., Kaczerowska-Pietrzak K., Florek E., Gornowicz-Porowska J., Pelant E., Nowak G. (2019). Theantiserotonin effect of parthenolide derivatives and standardized extract from the leaves of *Stizolophusbalsamita*. Molecules.

[43] Saeedi-Boroujeni A., Mahmoudian-Sani M.R. (2021). Anti-inflammatory potential of Quercetin in COVID-19 treatment. J. Inflamm..

[44] Diniz L.R.L., Filho C.S.M.B., Fielding B.C., de Sousa D.P. (2020). Natural Antioxidants: A Review of Studies on Human and Animal Coronavirus. Oxidative Med. Cell. Longev..

[45] Diniz L.R.L., Souza M.T., Duarte A.B.S.D., Sousa D.P. (2020). Mechanistic Aspects and Therapeutic Potential of Quercetin against COVID-19-Associated Acute Kidney Injury. Molecules.

[46] Darband S.G., Kaviani M., Yousefi B., Sadighparvar S., Pakdel F.G., Attari J.A., Mohebbi I., Naderi S., Majidinia M. (2018). Quercetin: A functional dietary flavonoid with potential chemo-preventive properties in colorectal cancer. J. Cell. Physiol..

[47] Terao J. (2017). Factors modulating bioavailability of quercetin-related flavonoids and the consequences of their vascular function. Biochem. Pharmacol..

[48] Kong L., Luo C., Li X., Zhou Y., He H. (2013). The anti-inflammatory effect of kaempferol on early atherosclerosis in high cholesterol fed rabbits. Lipids Health Dis..

[49] Imran M., Rauf A., Shah Z.A., Saeed F., Imran A., Arshad M.U., Ahmad B., Bawazeer S., Atif M., Peters D.G. (2019). Chemo-preventive and therapeutic effect of the dietary flavonoid kaempferol: A comprehensive review. Phytother. Res..

[50] Chen A.Y., Chen Y.C. (2013). A review of the dietary flavonoid, kaempferol on human health and cancer chemoprevention. Food Chem..

[51] Belchor N.M., Gaeta H.H., Rodrigues C.F.B., Costa C.R., Toyama D., Passero L.F.D., Laurenti M.D., Toyama M.H. (2017). Evaluation of Rhamnetin as an Inhibitor of the Pharmacological Effect of Secretory Phospholipase A2. Molecules.

[52] Majewska M., Skrzycki M., Podsiad M., Czeczot H. (2011). Evaluation of antioxidant potential of flavonoids: An in vitro study. Acta Pol. Pharm. Drug Res..

[53] Dok-Go H., Lee K.H., Kim H.J., Lee E.H., Lee J., Song Y.S., Lee Y.H., Jin C., Lee Y.S., Cho J. (2003). Neuroprotective effects of antioxidative flavonoids, quercetin, (+)-dihydroquercetin and quercetin 3-methyl ether, isolated from *Opuntiaficus-indica* var. *saboten*. Brain Res..

[54] Manigandan K., Manimaran D., Jayaraj R.L., Elangovan N., Dhivya V., Kaphle A. (2015). Taxifolin curbs NF-kappaB-mediated Wnt/beta-catenin signaling via up-regulating Nrf2 pathway in experimental colon carcinogenesis. Biochimie.

[55] Galochkina A.V., Anikin V.B., Babkin V.A., Ostrouhova L.A., Zarubaev V.V. (2016). Virus-inhibiting activity of dihydroquercetin, a flavonoid from *Larixsibirica*, against coxsackievirus B4 in a model of viral pancreatitis. Arch. Virol..

[56] Cai C., Liu C.H., Zhao L., Liu H., Li W., Guan H., Zhao L., Xiao J. (2018). Effects of Taxifolin on Osteoclastogenesis invitro and in vivo. Front. Pharmacol..

[57] Rodríguez P., González-Mujica F., Bermúdez J., Hasegawa M. (2010). Inhibition of glucose intestinal absorption by kaempferol 3-O-α-rhamnoside purified from Bauhinia megalandra leaves. Fitoterapia.

[58] Diantini A., Subarnas A., Lestari K., Lestari K., Halimah E., Susilawati Y., Supriyatna S., Julaeha E., Achmad T.H., Suradji E.W. (2012). Kaempferol-3-O-rhamnoside isolated from the leaves of SchimawallichiiKorth. inhibits MCF-7 breast cancer cell proliferation through activation of the caspase cascade pathway. Oncol. Lett..

[59] Sharoar M.G., Thapa A., Shahnawaz M., Ramasamy V.S., Woo E.-R., Shin S.Y., Park I.-S. (2012). Kaempferol-3-O-rhamnoside abrogates amyloid-beta toxicity by modulating monomers and remodeling oligomers and fibrils to non-toxic aggregates. J. Biomed. Sci..

[60] Báthori M., Pongrácz Z. (2005). Phytoecdysteroids From Isolation to Their Effects on Humans. Curr. Med. Chem..

[61] Gorelick-Feldman J., MacLean D., Ilic N., Poulev A., Lila M.A., Cheng D., Raskin I. (2008). Phytoecdysteroids increase protein synthesis in skeletal muscle cells. J. Agric. Food Chem..

[62] Dinan L. (2009). The Karlson lecture. Phytoecdysteroids: What use are they?. Arch. Insect Biochem. Physiol..

[63] Napierała M., Nawrot J., Gornowicz-Porowska J., Florek E., Moroch A., Adamski Z., Kroma A., Miechowicz I., Nowak G. (2020). Separation and HPLC characterisation natural steroids and a standardised extract from the *Serratulacoronata* herb with antiseborrheic dermatitis activity. Int. J. Environ. Res. Public Health.

[64] Migas P., Krauze-Baranowska M. (2015). The significance of arbutin and its derivatives in therapy and cosmetics. Phytochem. Lett..

[65] Balkrishnan R., Kelly A.P., Mc Michael A., Torok H. (2004). Improved quality of life with effective treatment of facial melasma: The pigment trial. J. Drugs Derm..

[66] Morag M., Nawrot J., Siatkowski I., Adamski Z., Fedorowicz T., Dawid-Pac R., Urbanska M., Nowak G. (2015). A double-blind, placebo-controlled randomized trial of *Serratulaequinquefoliae* folium, a new source of β-arbutin, in selected skin hyperpigmentations. J. Cosmet. Derm..

[67] Wagenitz G., Dittrich M., Rechinger K.H., Petrak F., Wagenitz G. (1980). Flora Iranica: Compositae III—Cynarae.

[68] Tutin T.G., Heywood V.H., Burges N.A., Moore D.M., Valentine D.H., Walters S.M., Weeb D.A. (1964). Flora Europaea.

