# Supplementary material for: Biologically Active Compounds in Stizolophus balsamita Inflorescences: Isolation, Phytochemical Characterization and Effects on the Skin Biophysical Parameters
Source: Int J Mol Sci. 2021 Apr 23;22(9):4428. doi: 10.3390/ijms22094428 (PMC8122880; doi:10.3390/ijms22094428)
Supplement: Supplementary file 1 [file ijms-22-04428-s001.zip › ijms-1161818-supplementary.pdf]

Biologically active compounds in Stizolophus balsamita inflorescences: isolation, phytochemical characterization and effects on the skin biophysical parameters

Joanna Nawrot[1], Jaromir Budzianowski[2], Gerard Nowak[1], Iwona Micek[1], Anna Budzianowska[2] and Justyna Gornowicz-Porowska[1]*

[1] Department and Division of Practical Cosmetology and Skin Diseases Prophylaxis, Poznan University of Medicinal Sciences, Mazowiecka 33, 60-623 Poznan, Poland
[2] Department of Pharmaceutical Botany and Plant Biotechnology, Poznan University of Medical Sciences, SwMariiMagdaleny Str., 60-356 Poznan, Poland

Supplementary material

List of supporting information

Compound **1** (izospiciformin).

Compound **2**(stizolin).

Compound **3**(stizolicin).

Compound **4**(quercetin).

Compound **5** (kaempferol).

Compound **6** (rhamnetin).

Compound **7**(taxifolin).

Compound **8** (kaempferol 3-*O*-α-rhamnoside).

Compound **9**(ajugasterone C).

Compound **10**(arbutin).

Compound **11** (protocatechuic acid methyl ester).

List of spectra:

Figure S1. ^{1}H NMR spectrum (600 MHz, CD_3OD) of compound **4**.

Figure S2. ^{1}H NMR spectrum (600 MHz, CD_3OD) of compound **5**.

Figure S3. EI-MS spectrum of compound **6**.

Figure S4. ^{1}H NMR spectrum (600 MHz, CD_3OD) of compound **7**.

Figure S5. ^{13}C NMR spectrum (150 MHz, CD_3OD) of compound **7**.

Figure S6. HH-COSY spectrum (600 MHz, CD_3OD) of compound **7**.

Figure S7. HSQC spectrum (600/150 MHz, CD_3OD) of compound **7**.

Figure S8. HMBC spectrum (600/150 MHz, CD_3OD) of compound **7**.

Figure S9. NOESY spectrum (600 MHz, CD_3OD) of compound **7**.

Figure S10. ^{1}H NMR spectrum (600 MHz, CD_3OD) of a mixture of compounds **8** and **9**. Only signals of compound **8** are labeled.

Figure S11. ^{13}C NMR spectrum (150 MHz, CD_3OD) of a mixture of compounds **8** and **9**.

Figure S12. ^{13}C NMR spectrum (150 MHz, CD$_3$OD) of a mixture of compounds **8** and **9**. Only signals of compound **8** are labeled.

Figure S13. HH-COSY spectrum (600 MHz, CD$_3$OD) of a mixture of compounds **8** and **9**. Only signals of compound **8** are labeled.

Figure S14. HSQC spectrum (600/150 MHz, CD$_3$OD) of a mixture of compounds **8** and **9**. Only signals of compound **8** are labeled.

Figure S15. HMBC spectrum (600/150 MHz, CD$_3$OD) of a mixture of compounds **8** and **9**. Only signals of compound **8** are labeled.

Figure S16. NOESY spectrum (600 MHz, CD$_3$OD) of a mixture of compounds **8** and **9**.

Figure S17. ^{1}H NMR spectrum (600 MHz, CD$_3$OD) of a mixture of compounds **7**, **10** and **11**.

Figure S18. ^{1}H NMR spectrum (600 MHz, CD$_3$OD) of a mixture of compounds **7**, **10** and **11**. Only signals of compound **11** are labeled.

Figure S19. ^{1}H NMR spectrum (600 MHz, CD$_3$OD) of a mixture of compounds **7**, **10** and **11**. Only signals of compound **10** are labeled.

Figure S20. ^{1}H NMR spectrum (600 MHz, CD$_3$OD) of a mixture of compounds **7**, **10** and **11**.Only signals of compound **7** are labeled.

Figure S21. ^{13}C NMR spectrum (150 MHz, CD$_3$OD) of a mixture of compounds **7**, **10** and **11**.

Figure S22. HH-COSY spectrum (600 MHz, CD$_3$OD) of a mixture of compounds **7**, **10** and **11**.

Figure S23. HSQC spectrum (600/150 MHz, CD$_3$OD) of a mixture of compounds **7**, **10** and **11**.

Figure S24. HMBC spectrum (600/150 MHz, CD$_3$OD) of a mixture of compounds **7**, **10** and **11**. The signals of compound **7** are labeled.

Figure S25. ^{1}H NMR spectrum (600 MHz, CD$_3$OD) of arbutin from *Serratulaquinquefolia*.

Figure S26. ^{13}C NMR spectrum (150 MHz, CD$_3$OD) of arbutin from *Serratulaquinquefolia*.

Figure S27. HH-COSY spectrum (600 MHz, CD$_3$OD) of arbutin from *Serratulaquinquefolia*.

Figure S28. HSQC spectrum (600/150 MHz, CD$_3$OD) of arbutin from *Serratulaquinquefolia*.

List of tables:

Table S1.

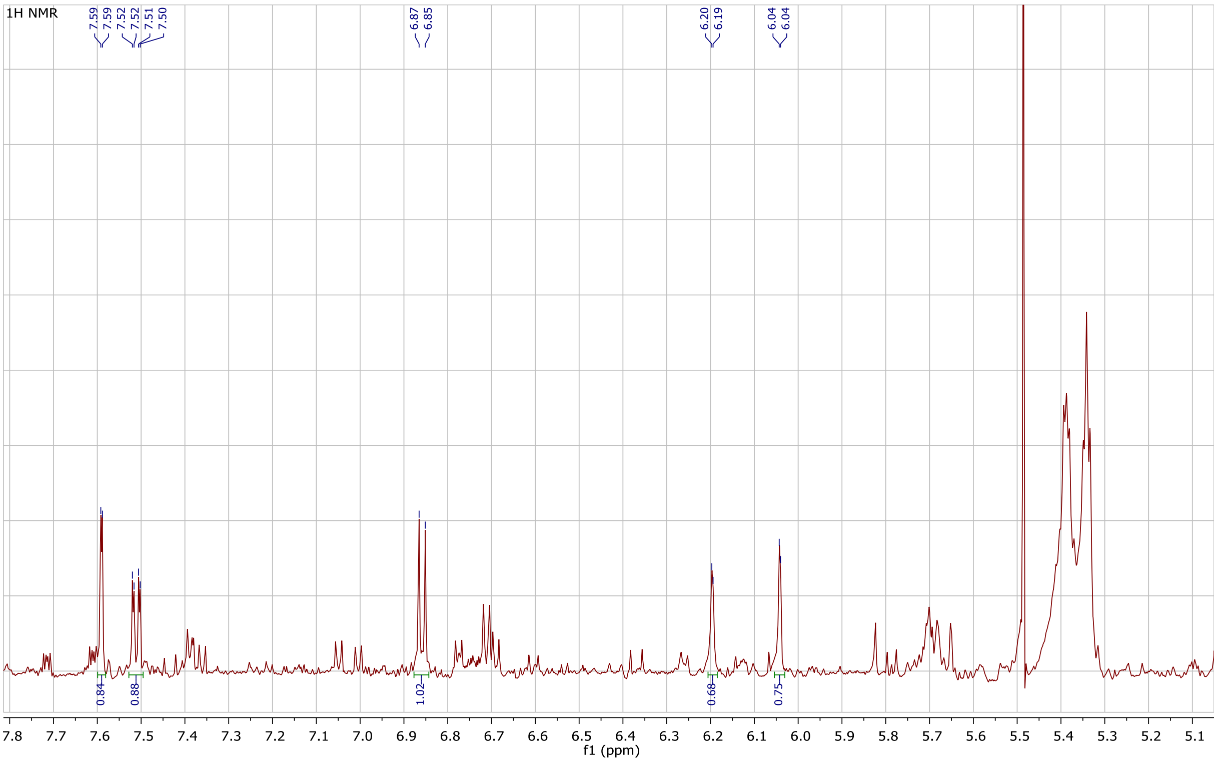

Figure S1. ^{1}H NMR spectrum (600 MHz, CD_3OD) of compound **4**.

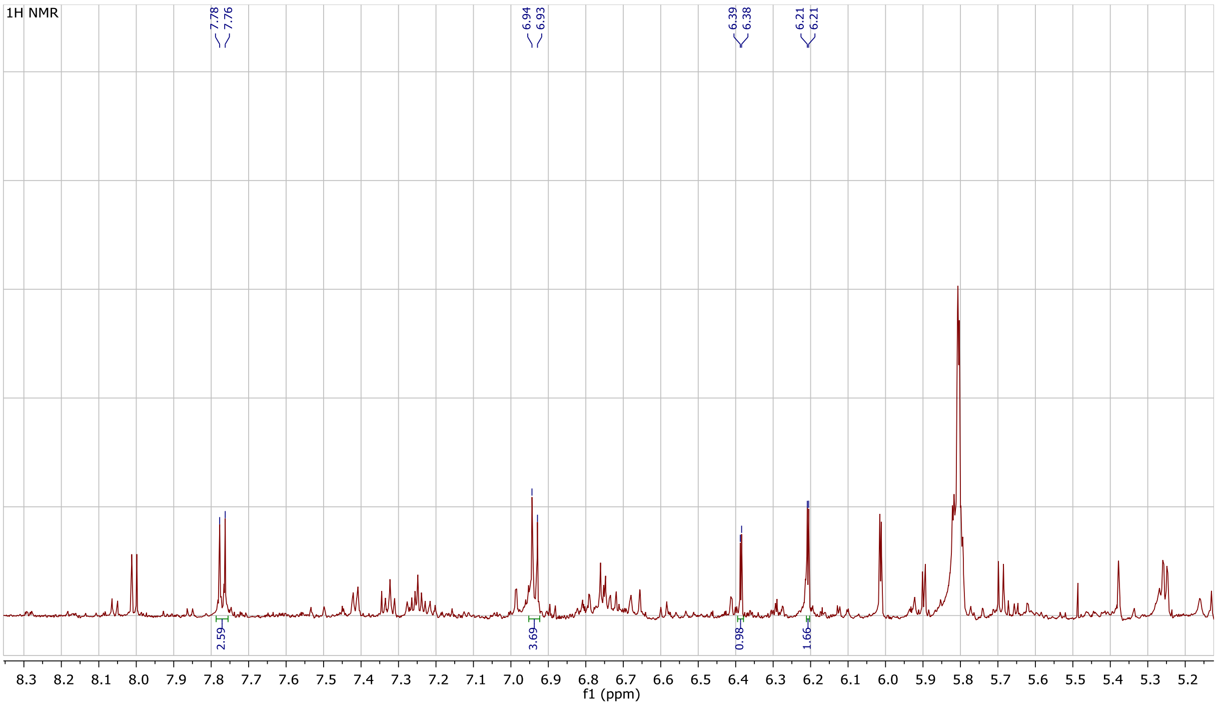

Figure S2. ^{1}H NMR spectrum (600 MHz, CD_3OD) of compound **5**.

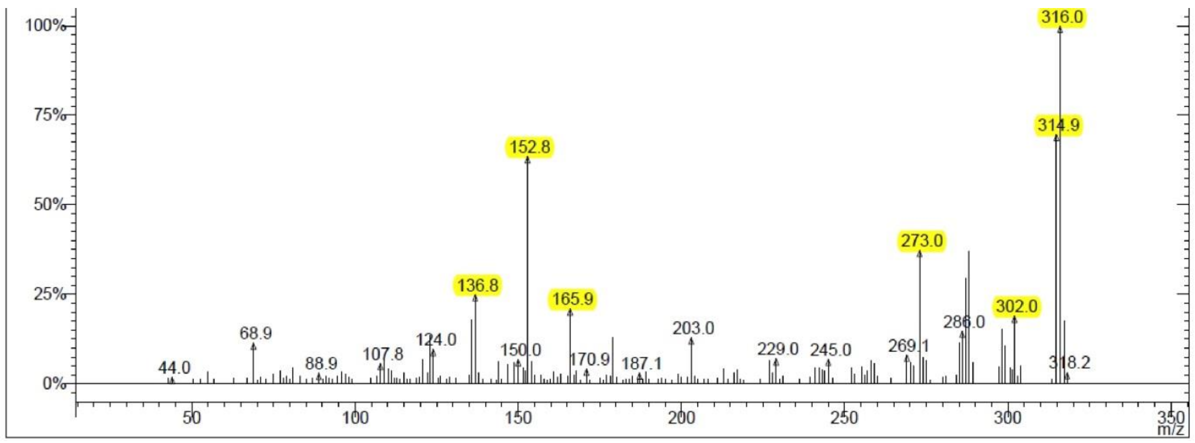

Figure S3. EI-MS spectrum of compound **6**.

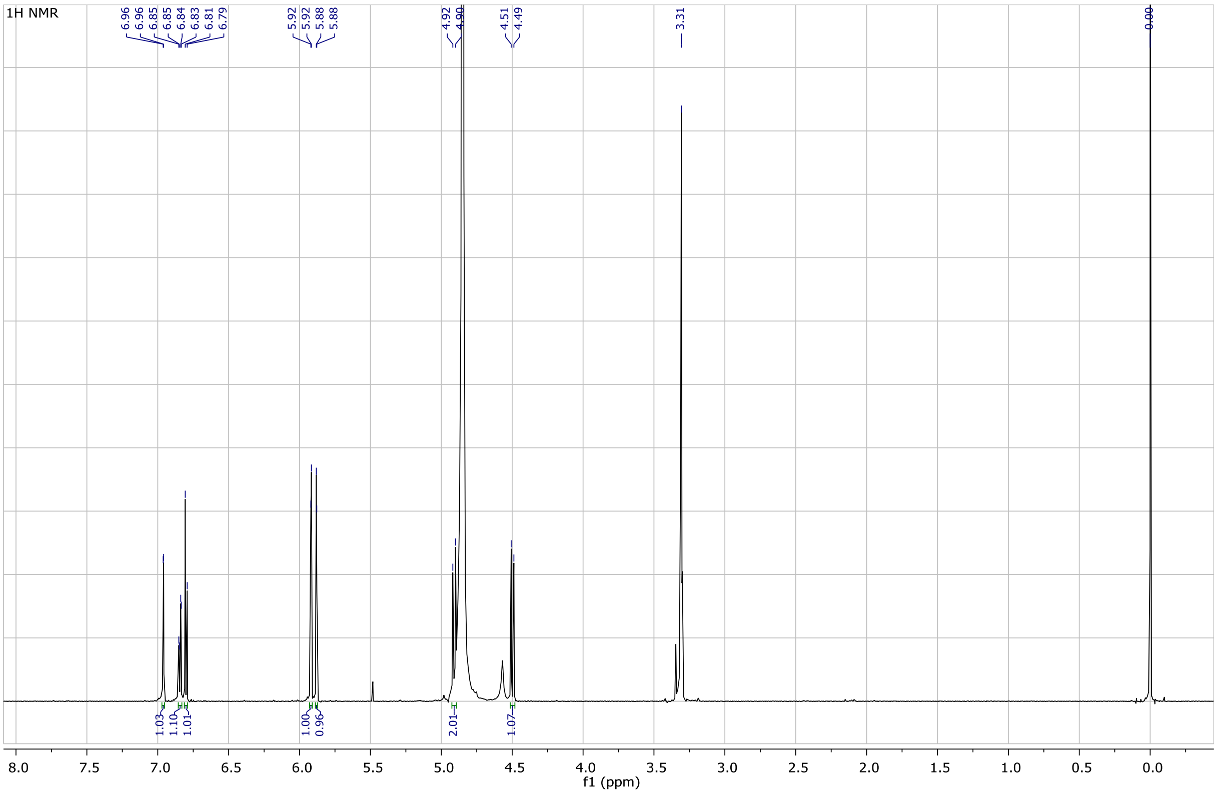

Figure S4. ^{1}H NMR spectrum (600 MHz, CD_3OD) of compound **7**.

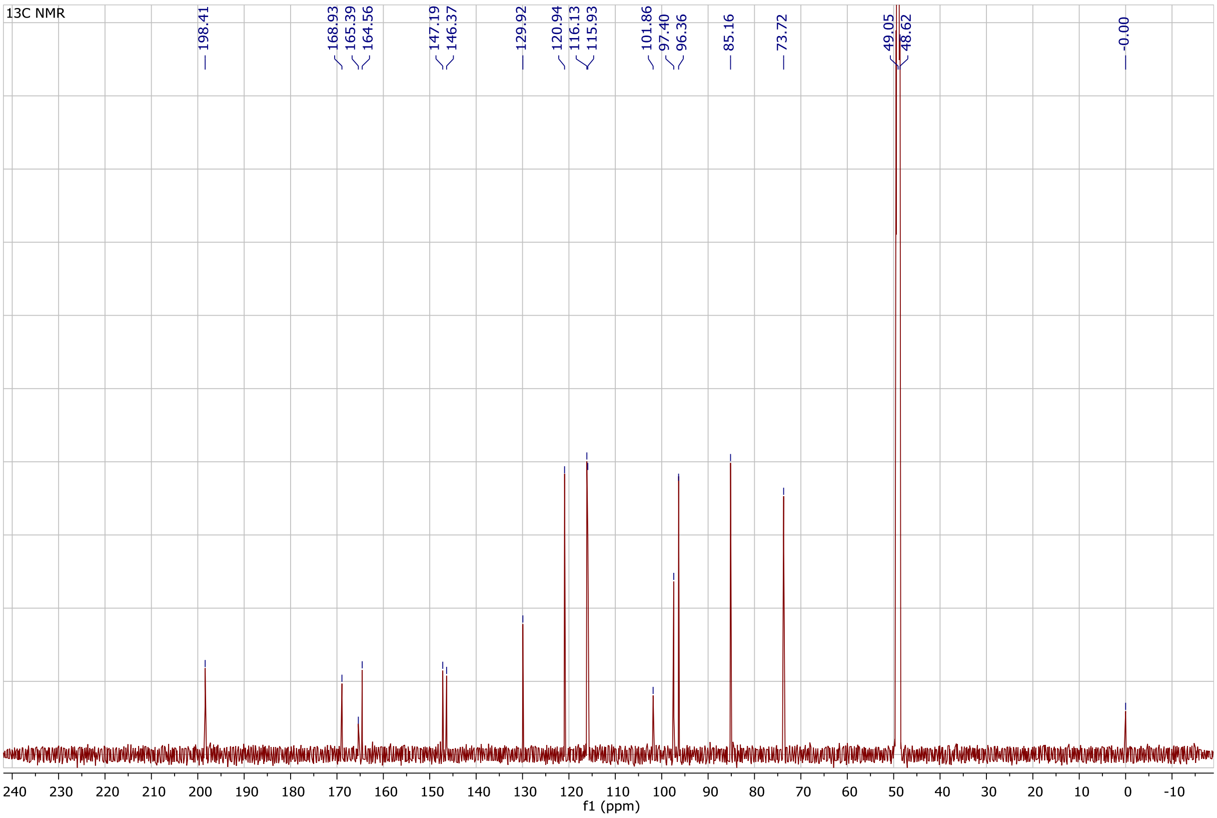

Figure S5. ^{13}C NMR spectrum (150 MHz, CD_3OD) of compound **7**.

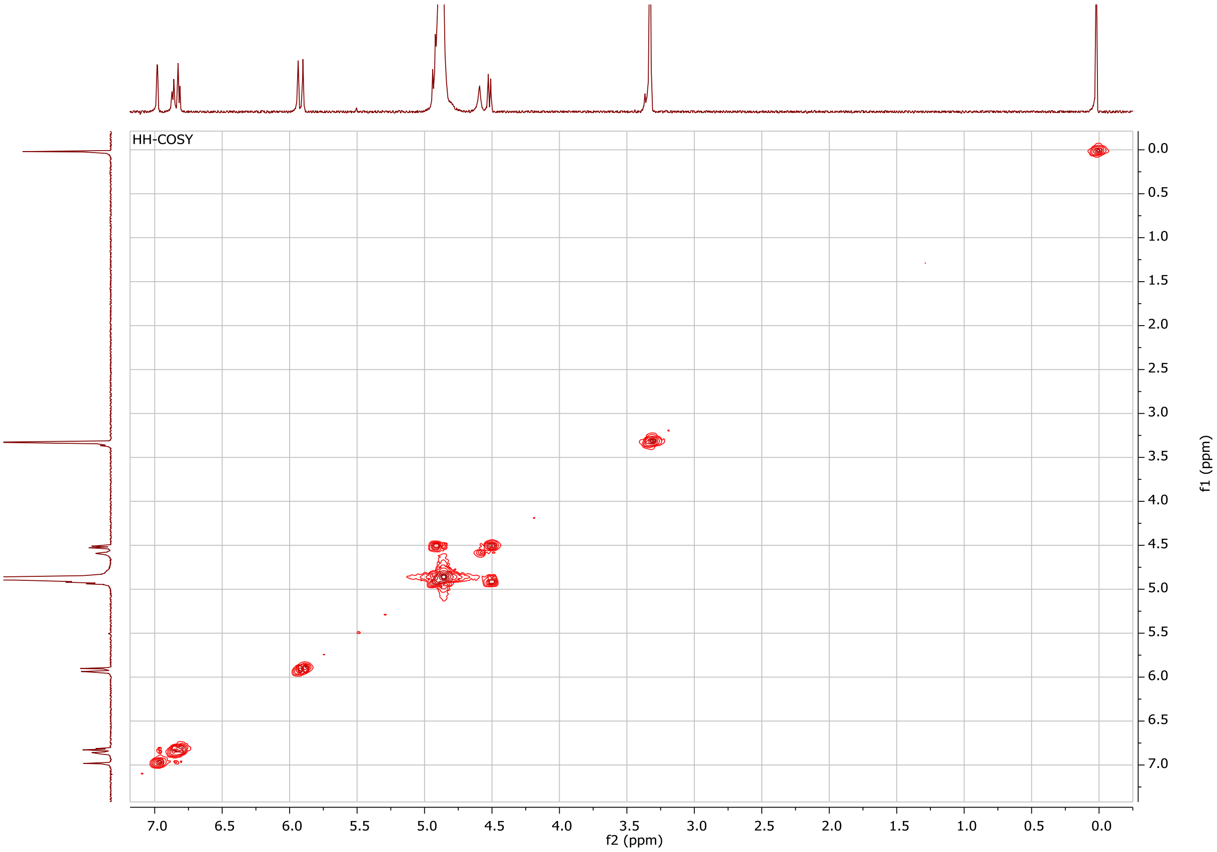

Figure S6. HH-COSY spectrum (600 MHz, CD_3OD) of compound **7**.

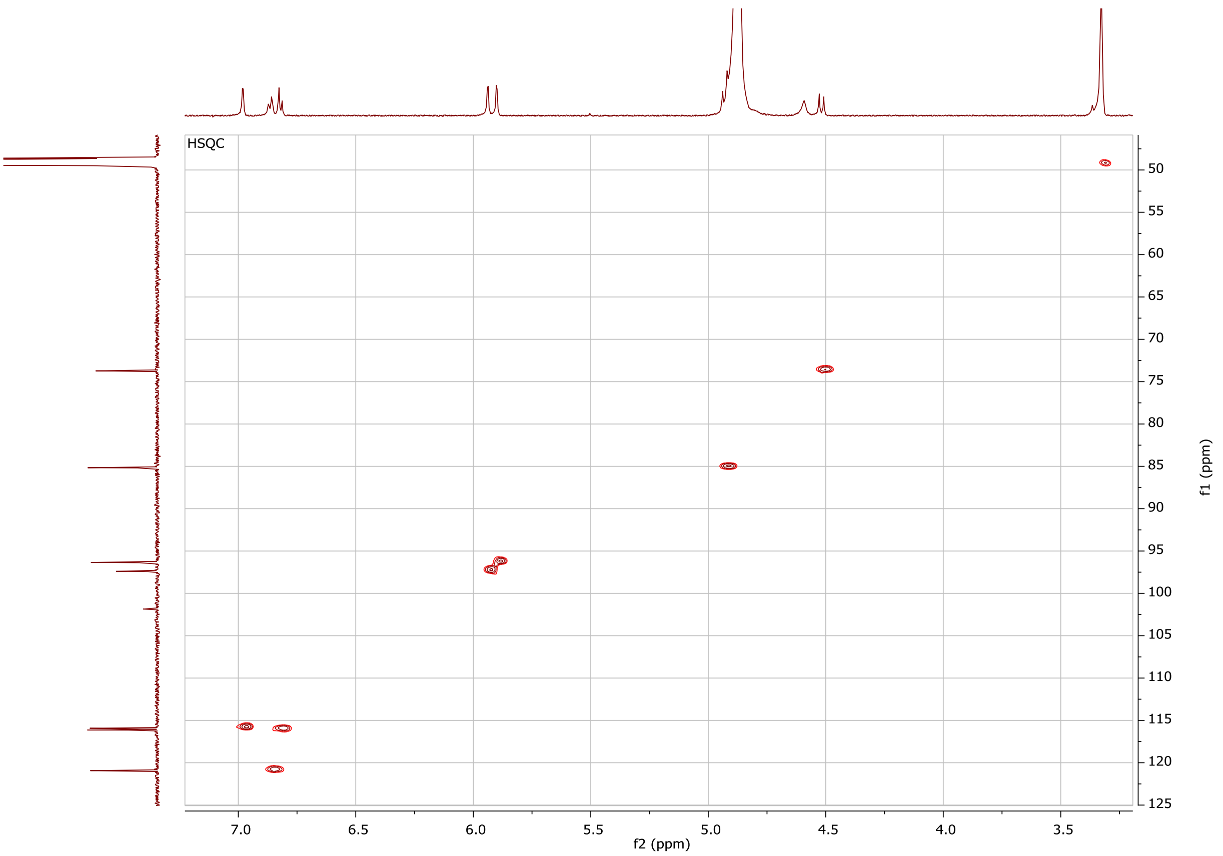

Figure S7. HSQC spectrum (600/150 MHz, CD_3OD) of compound **7**.

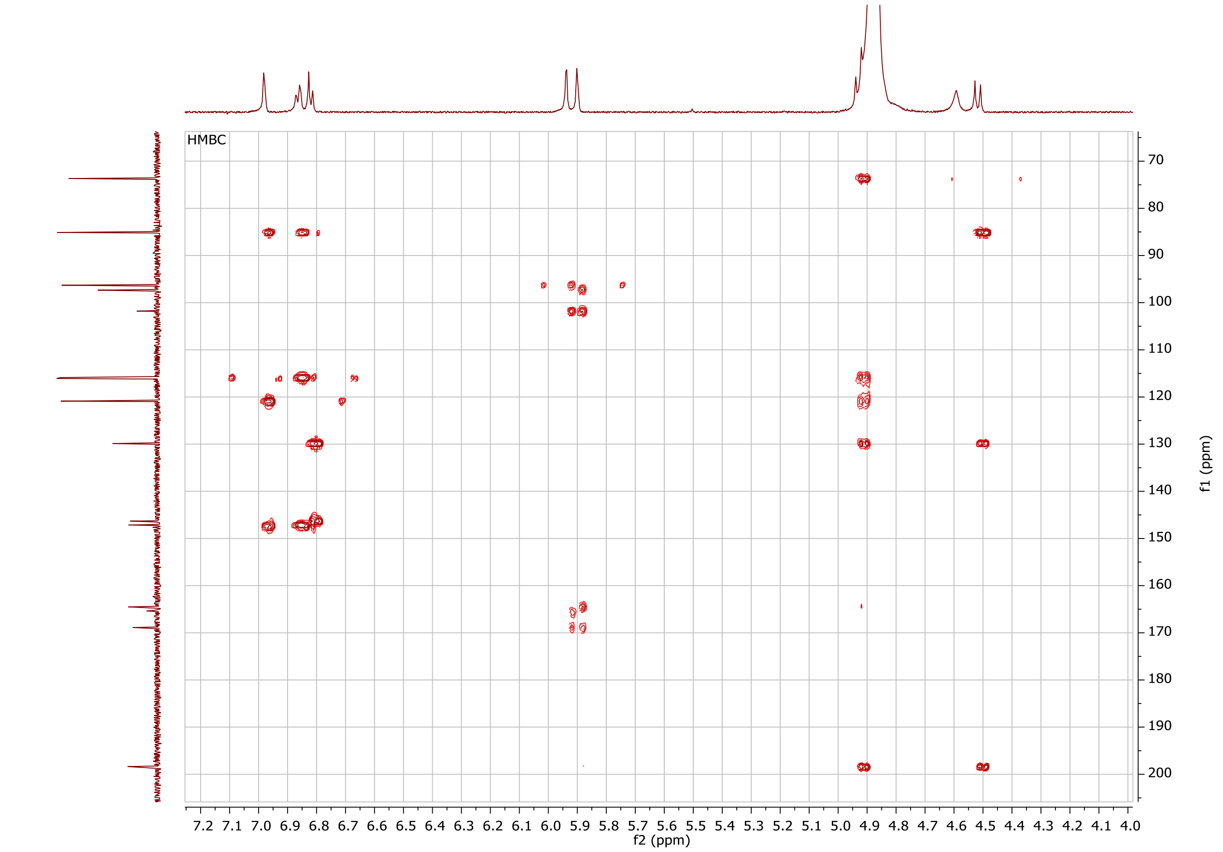

Figure S8. HMBC spectrum (600/150 MHz, CD_3OD) of compound **7**.

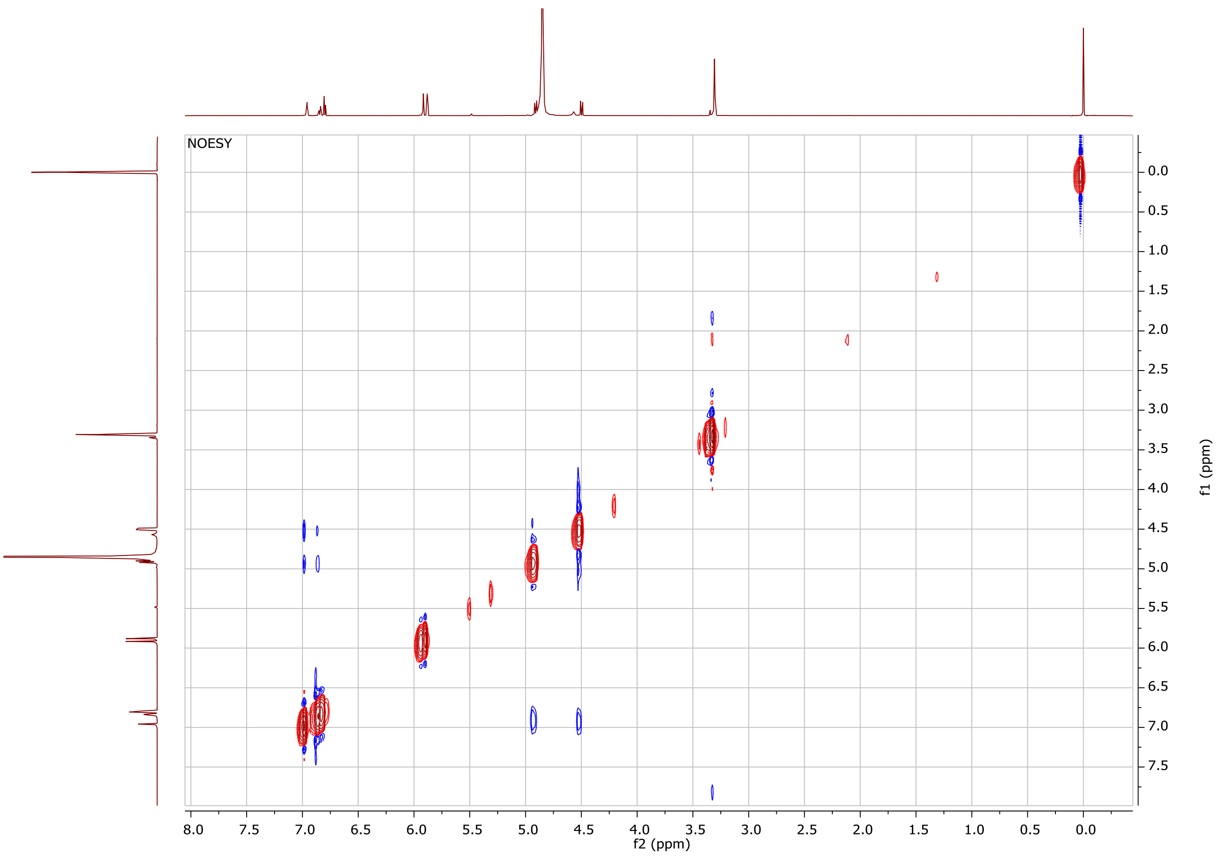

Figure S9. NOESY spectrum (600 MHz, CD_3OD) of compound **7**.

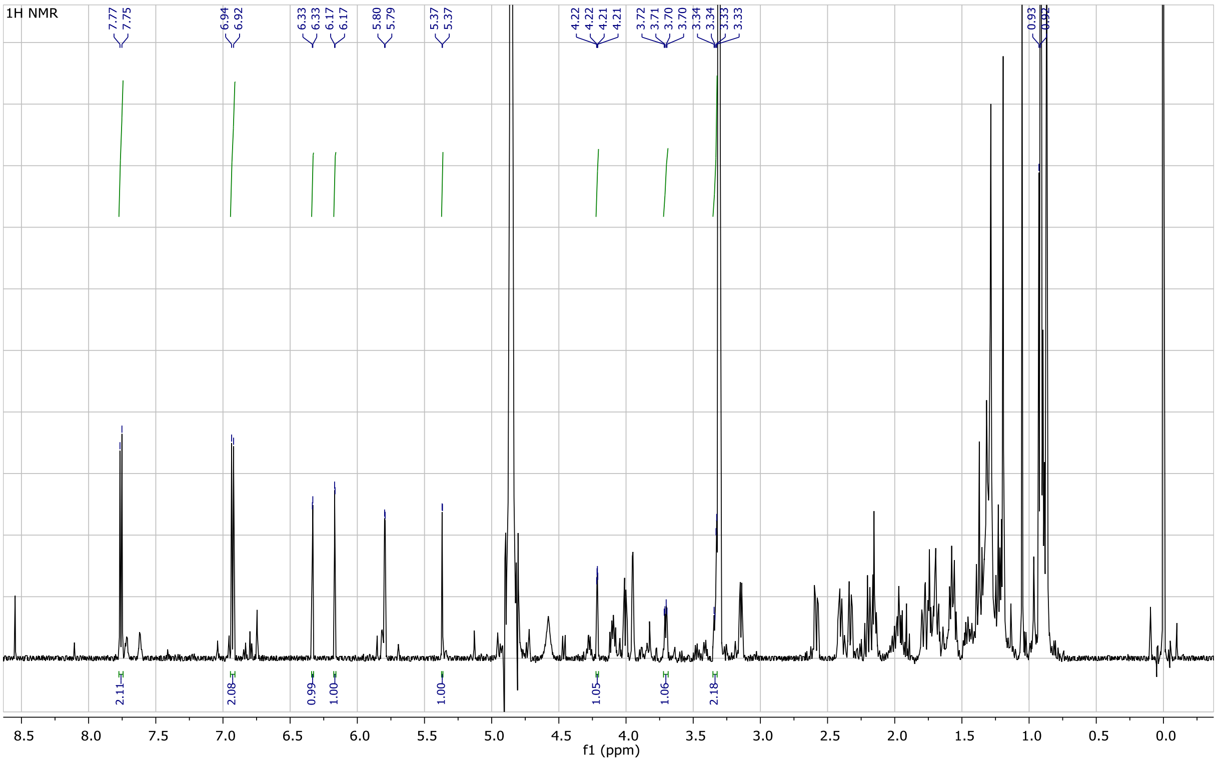

Figure S10. ^1H NMR spectrum (600 MHz, CD_3OD) of a mixture of compounds**8**and**9**. Only signals of compound **8** are labeled.

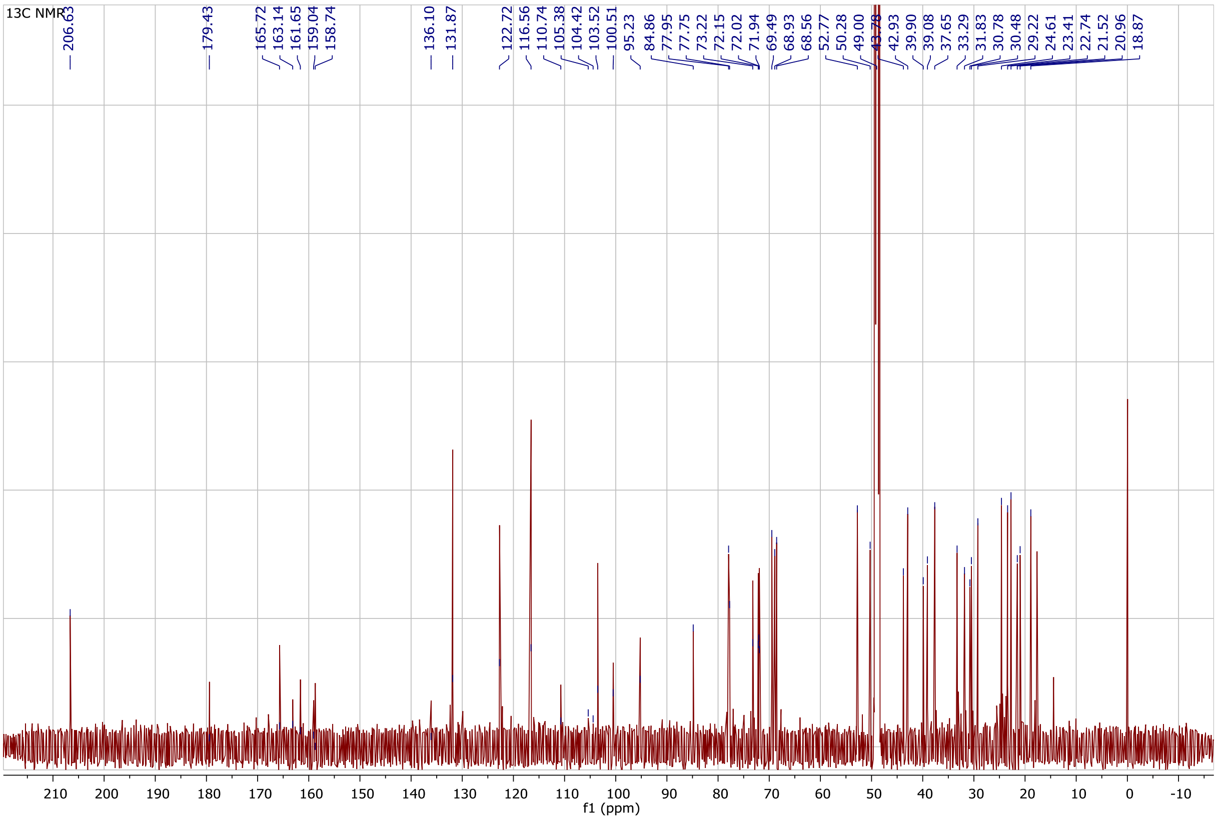

Figure S11. ^{13}C NMR spectrum (150 MHz, CD_3OD) of a mixture of compounds**8** and**9**.

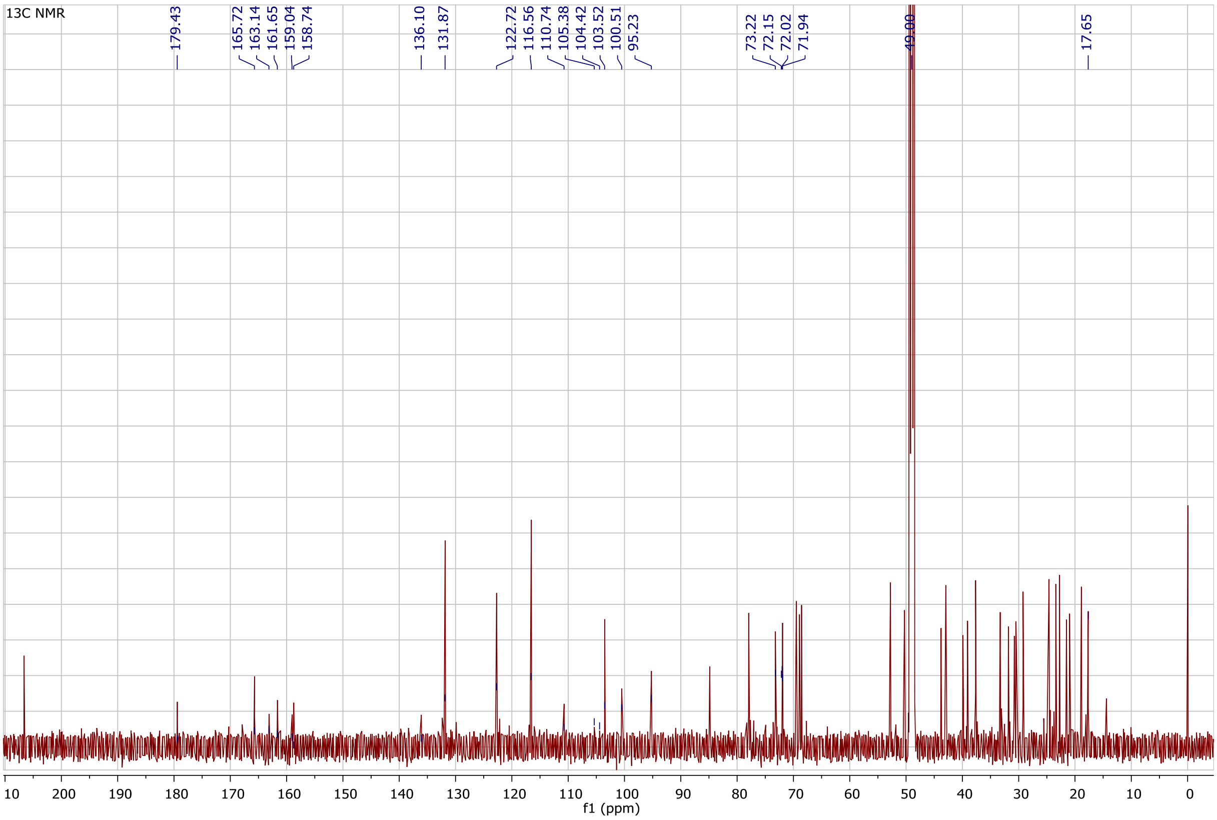

Figure S12. ^{13}C NMR spectrum (150 MHz, CD_3OD) of a mixture of compounds**8** and**9**. Only signals of compound **8** are labeled.

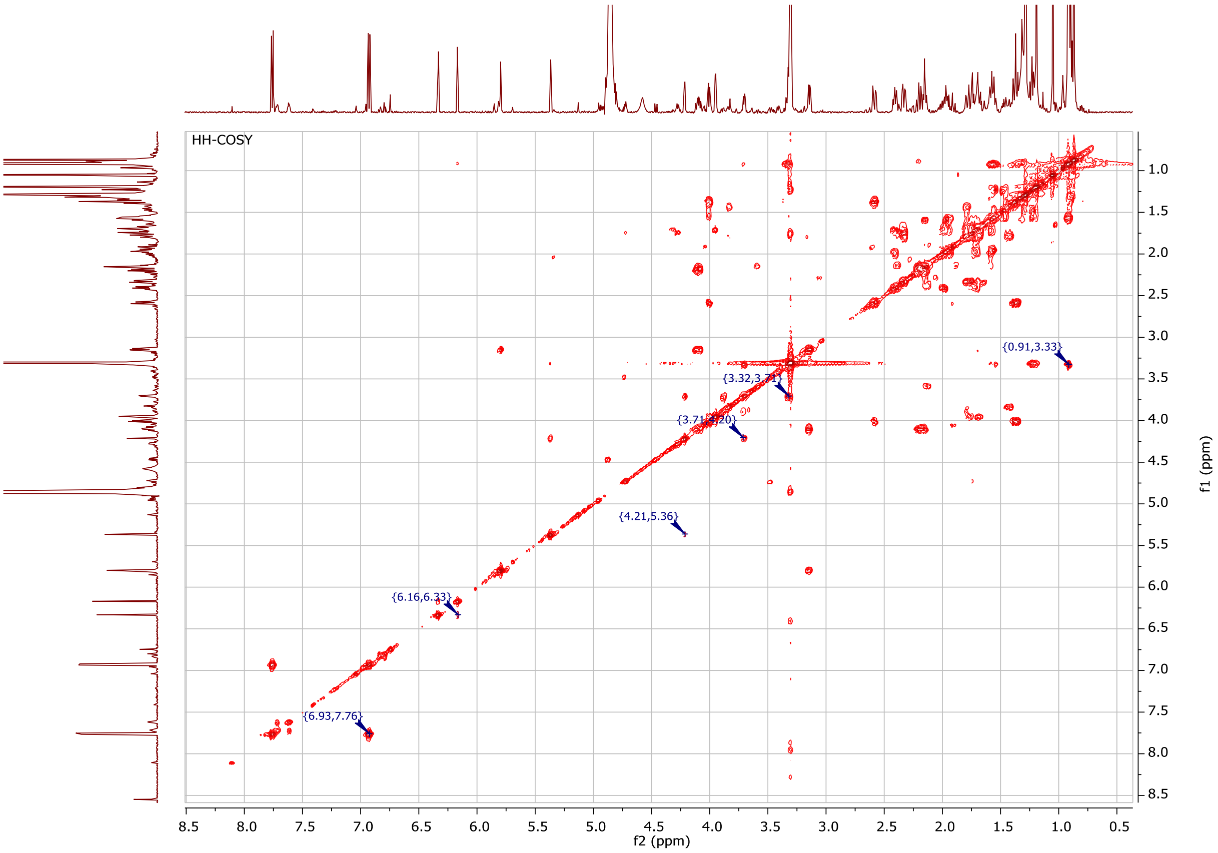

Figure S13. HH-COSY spectrum (600 MHz, CD_3OD) of a mixture of compounds**8** and**9**. Only signals of compound **8** are labeled.

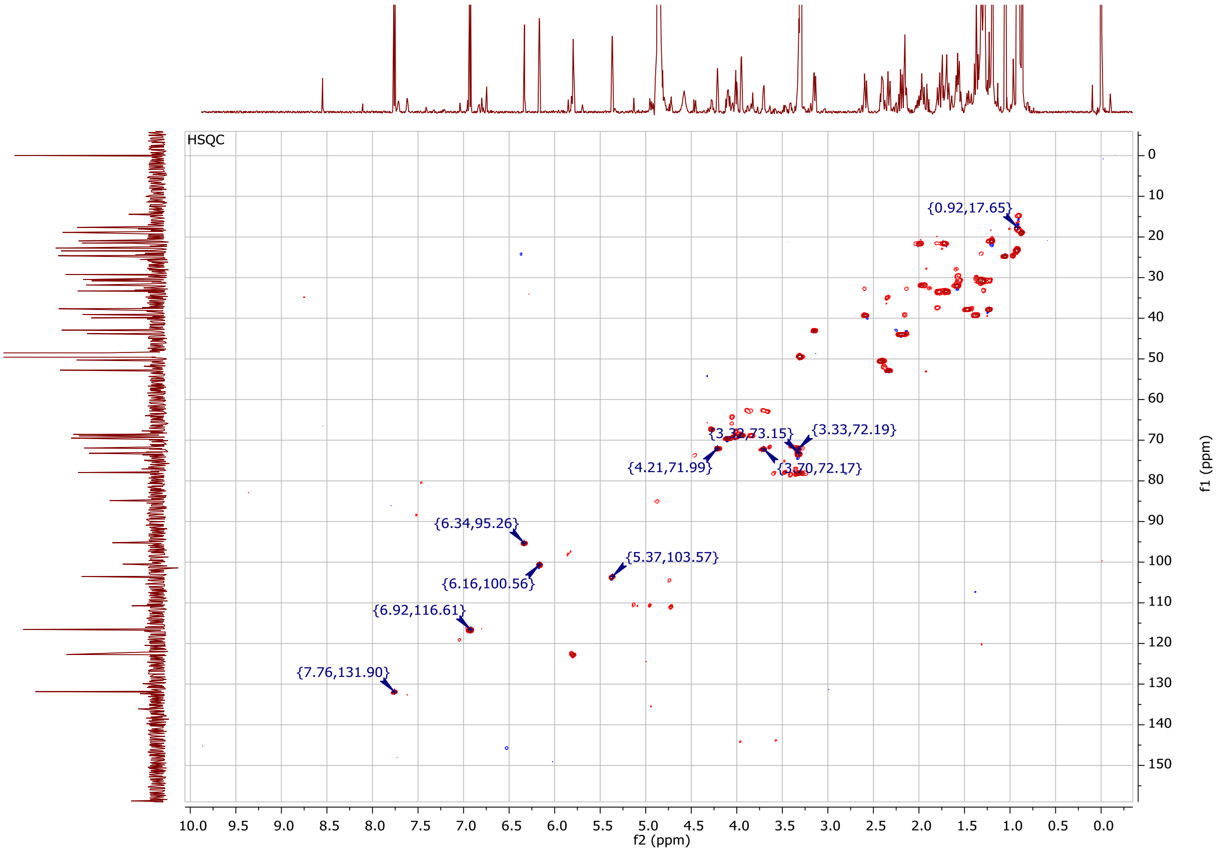

Figure S14. HSQC spectrum (600/150 MHz, CD_3OD) of a mixture of compounds**8** and**9**. Only signals of compound **8** are labeled.

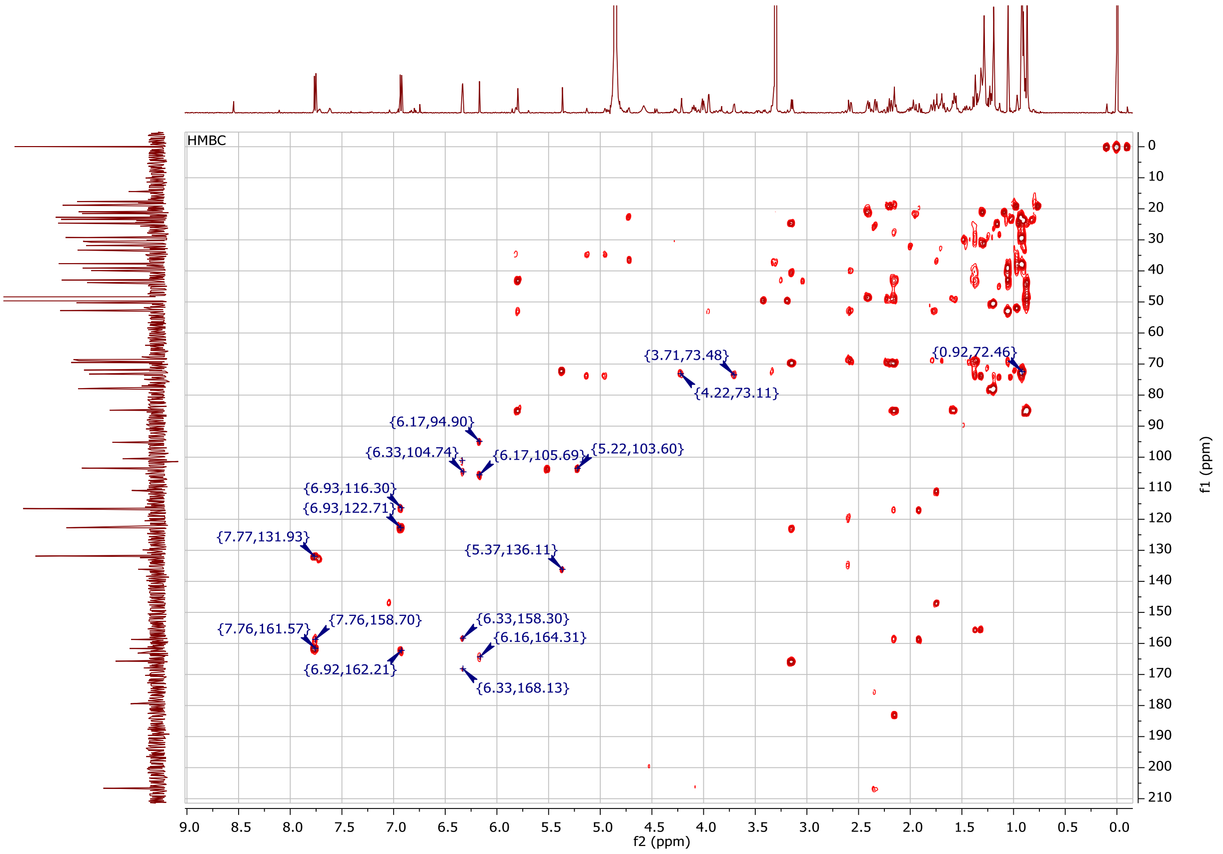

Figure S15. HMBC spectrum (600/150 MHz, CD_3OD) of a mixture of compounds**8** and**9**. Only signals of compound **8** are labeled.

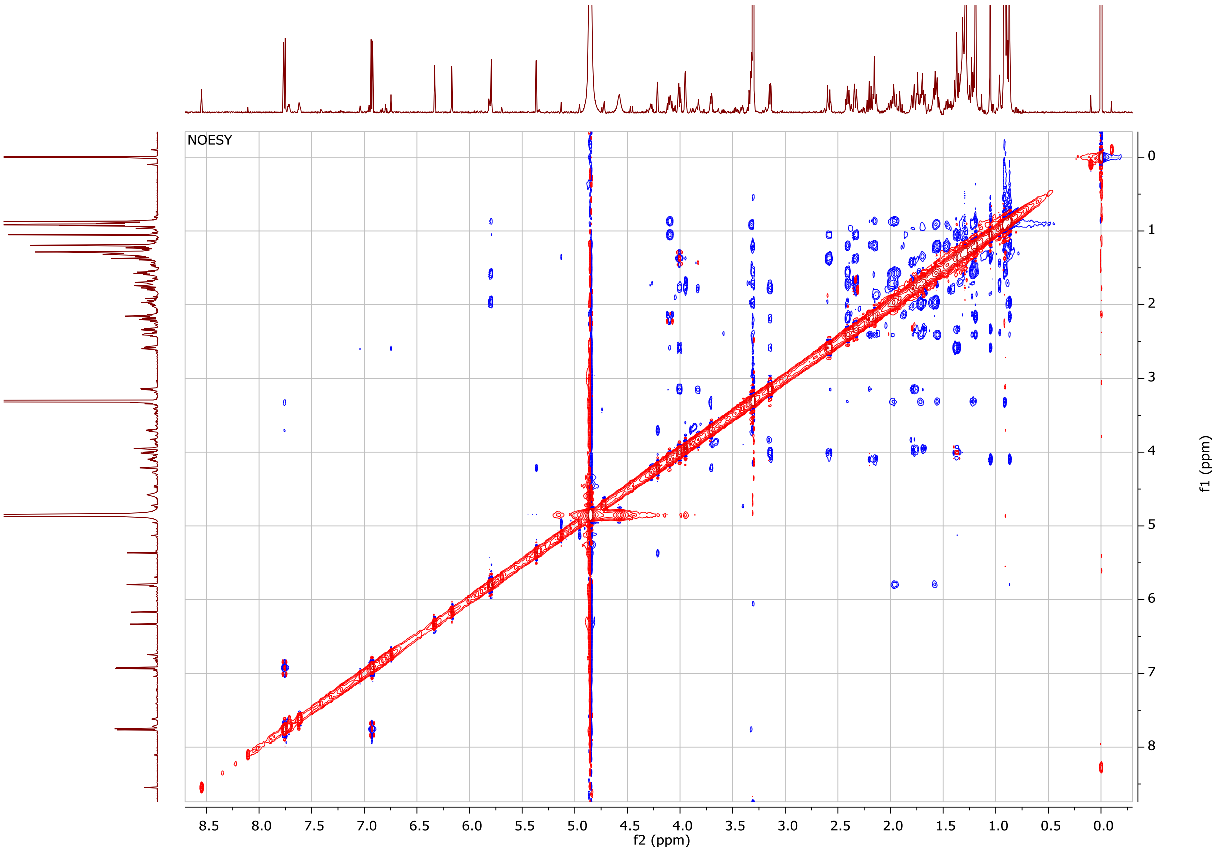

Figure S16. NOESY spectrum (600 MHz, CD_3OD) of a mixture of compounds **8** and**9**.

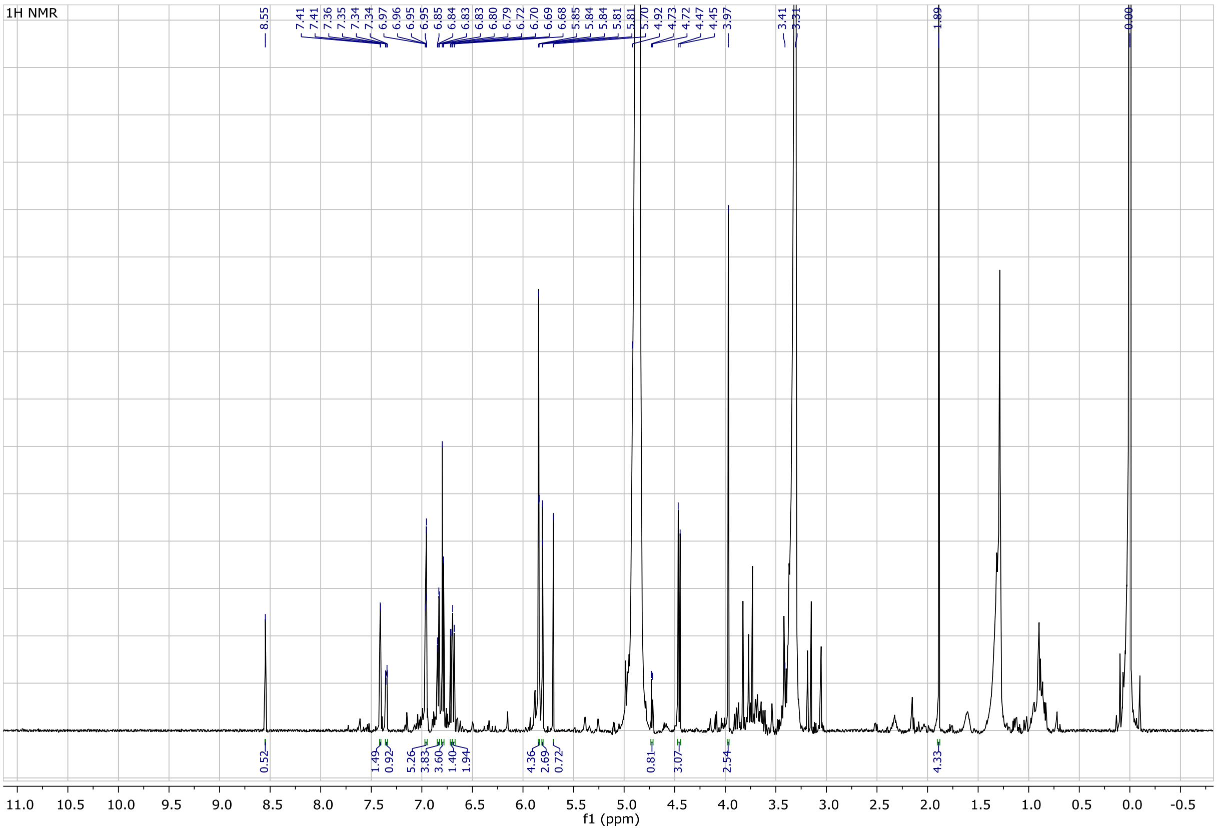

Figure S17. ^{1}H NMR spectrum (600 MHz, CD_3OD) of a mixture of compounds**7**, **10** and**11**.

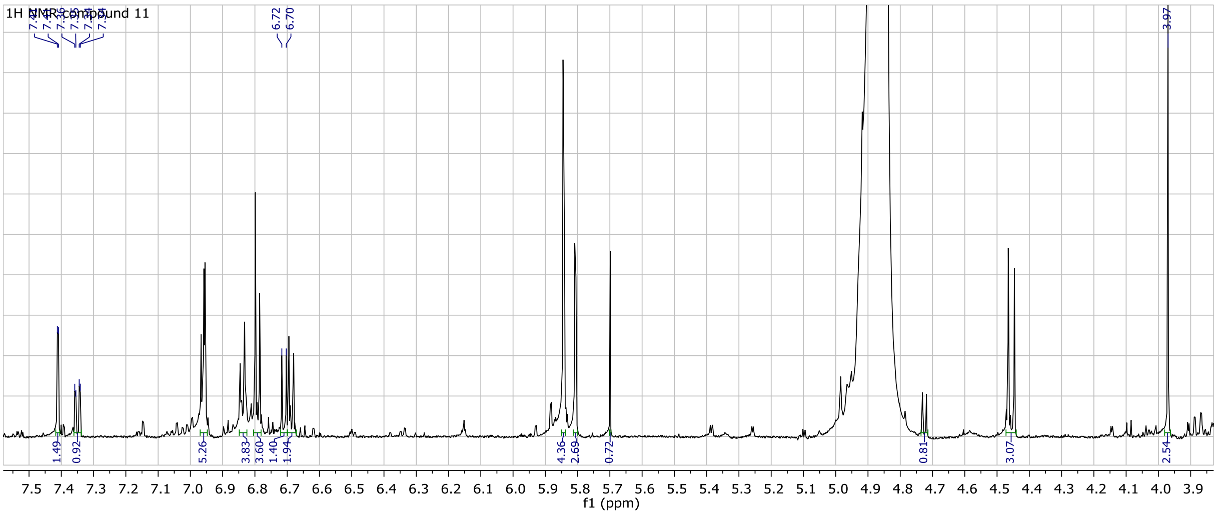

Figure S18. ^{1}H NMR spectrum (600 MHz, CD_3OD) of a mixture of compounds**7**, **10** and**11**.Only signals of compound **11** are labeled.

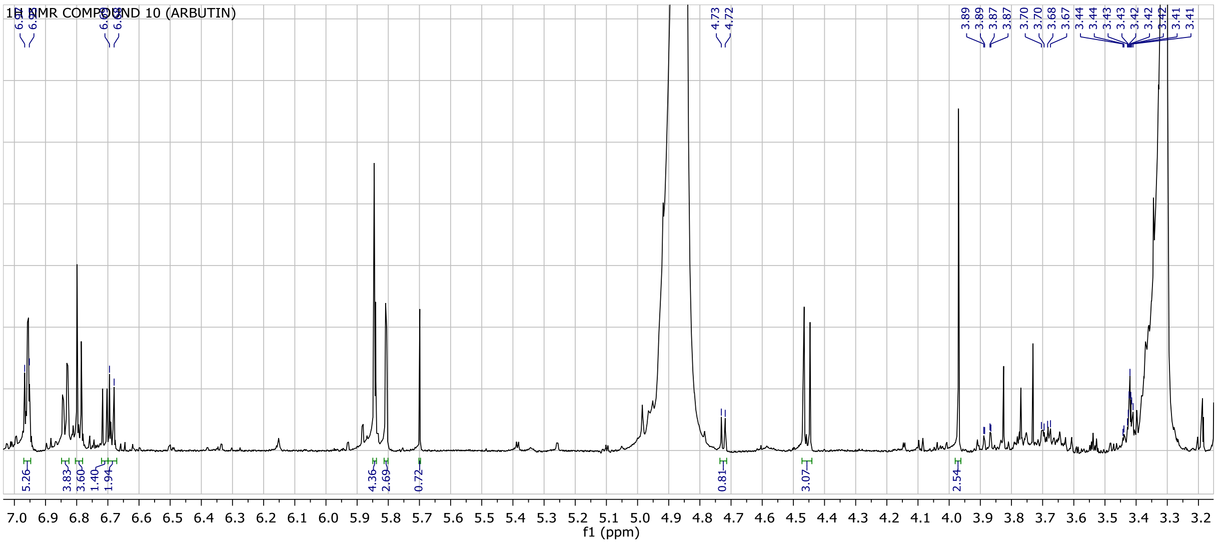

Figure S19. ^{1}H NMR spectrum (600 MHz, CD_3OD) of a mixture of compounds**7**, **10** and**11**. Only signals of compound **10** are labeled.

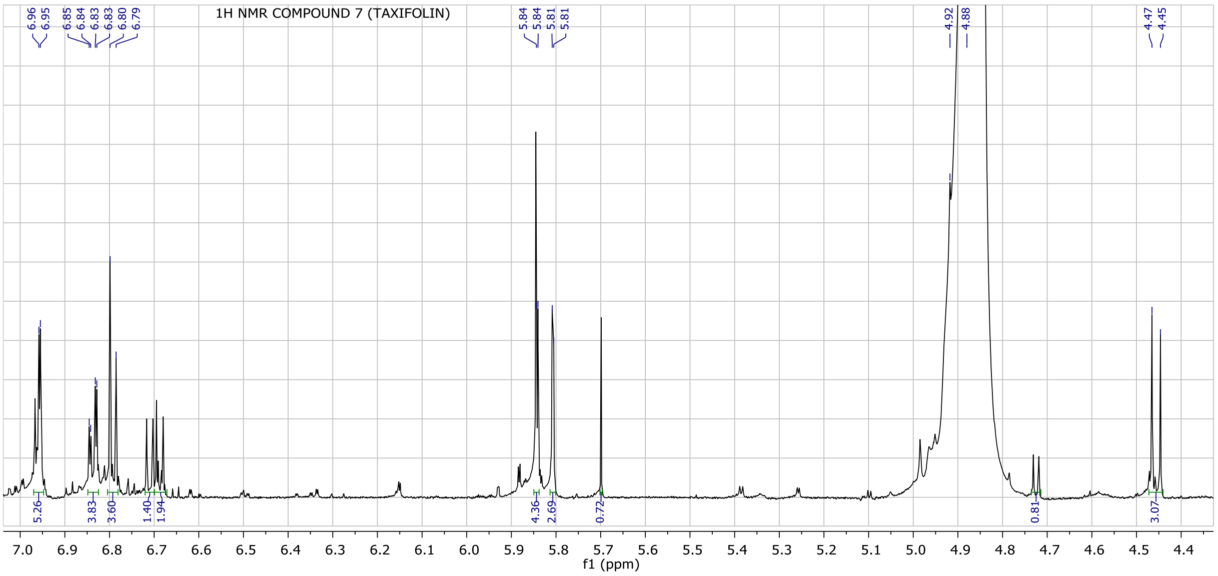

Figure S20. ^{1}H NMR spectrum (600 MHz, CD_3OD) of a mixture of compounds**7**, **10** and**11**.Only signals of compound **7** are labeled.

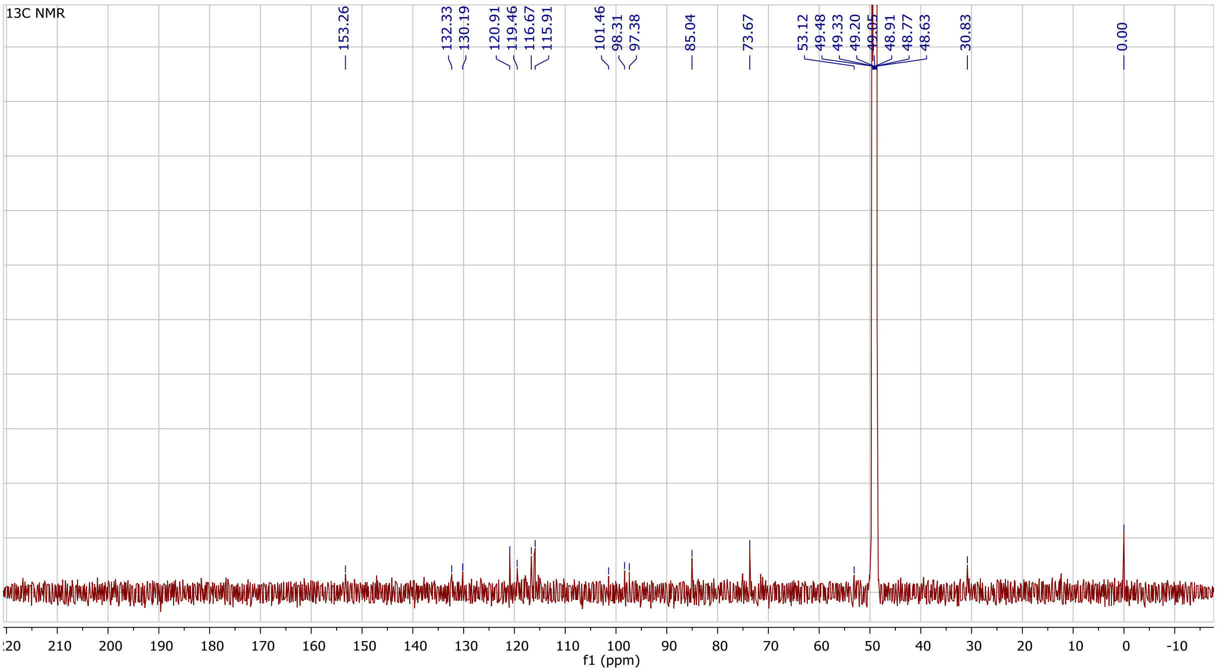

Figure S21. ^{13}C NMR spectrum (150 MHz, CD_3OD) of a mixture of compounds**7**, **10** and**11**.

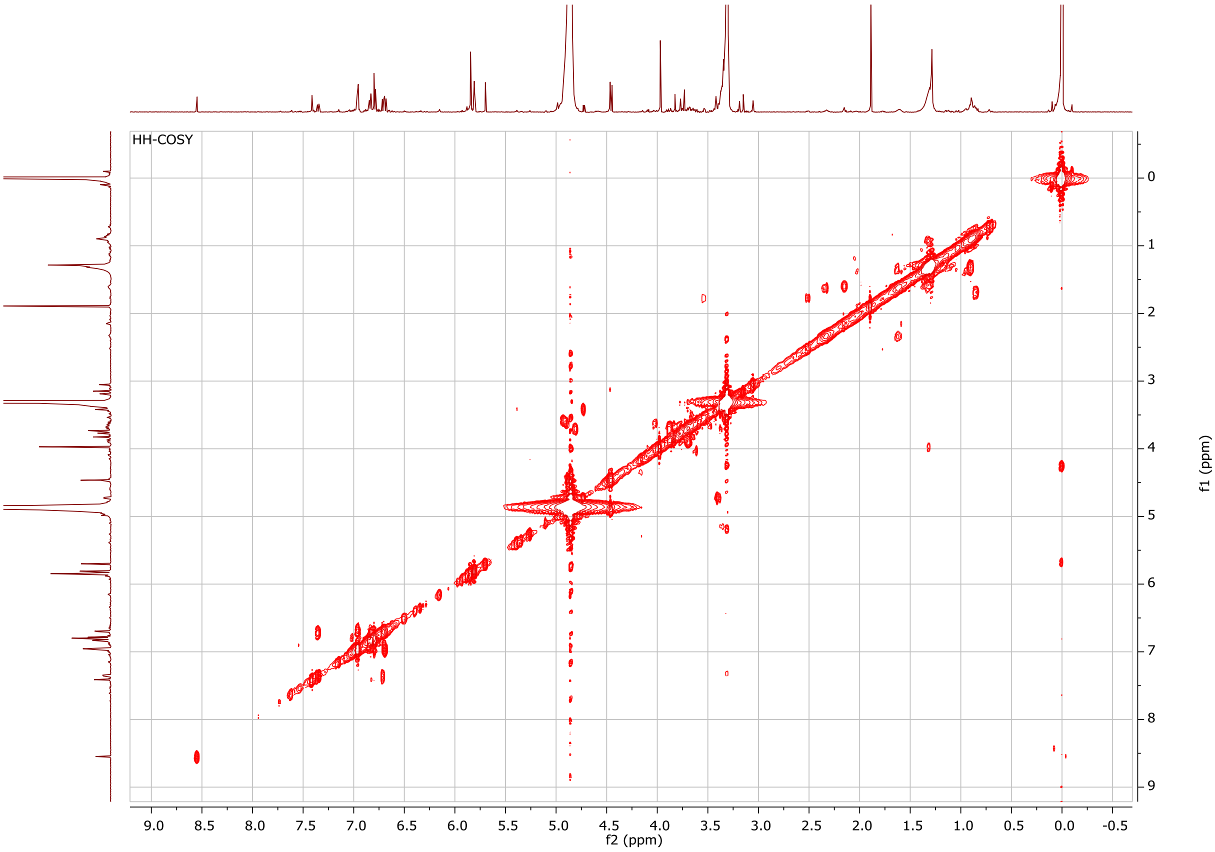

Figure S22. HH-COSY spectrum (600 MHz, CD_3OD) of a mixture of compounds**7**, **10** and**11**.

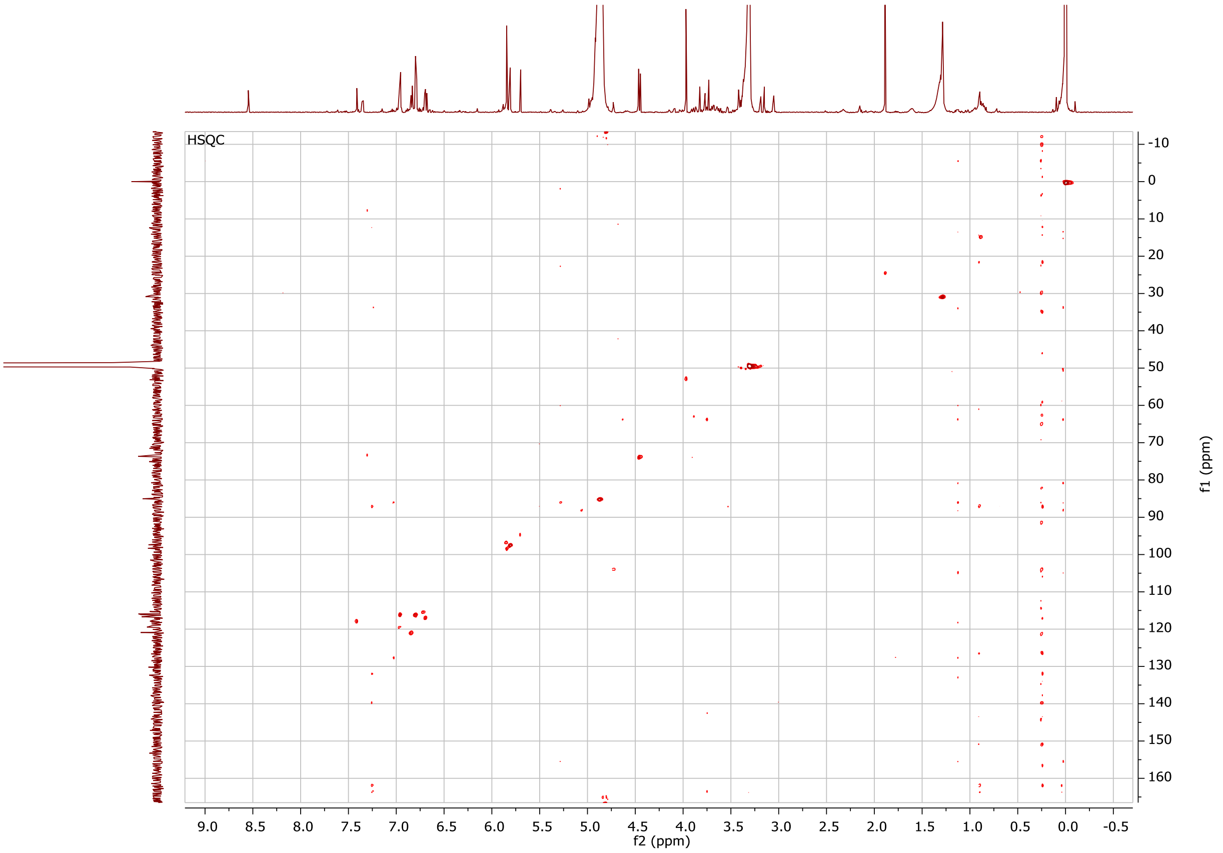

Figure S23. HSQC spectrum (600/150 MHz, CD_3OD) of a mixture of compounds**7**, **10** and**11**.

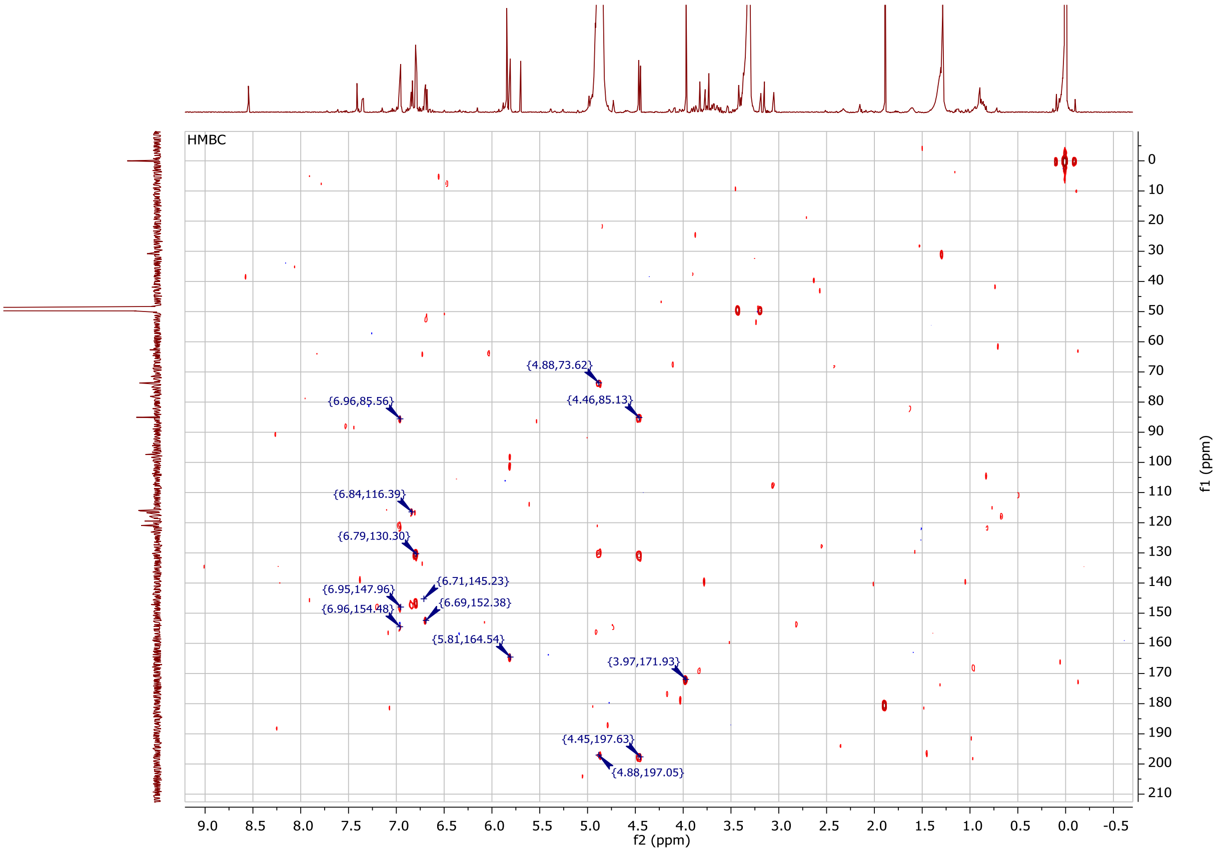

Figure S24. HMBC spectrum (600/150 MHz, CD_3OD) of a mixture of compounds**7**, **10** and**11**. The signals of compound **7** are labeled.

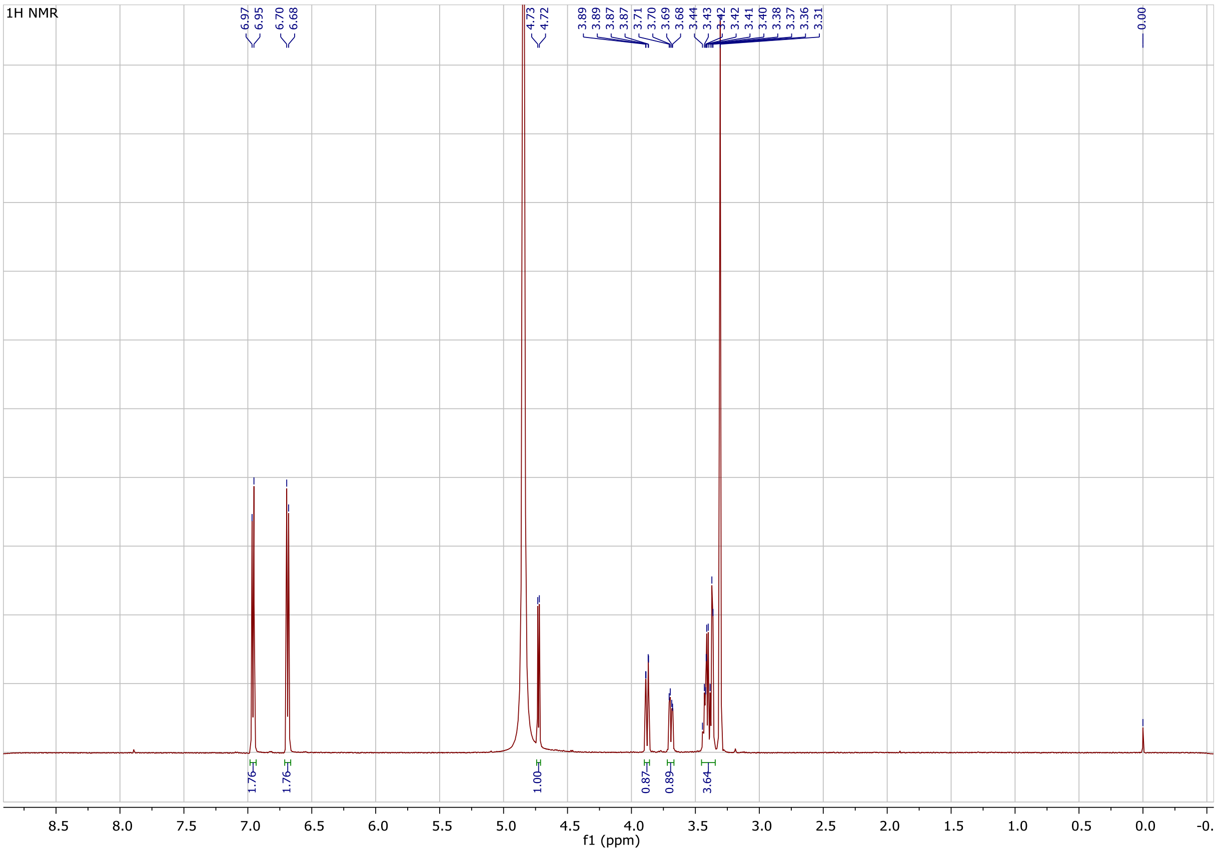

Figure S25. ^{1}H NMR spectrum (600 MHz, CD_3OD) of arbutin from *Serratulaquinquefolia*.

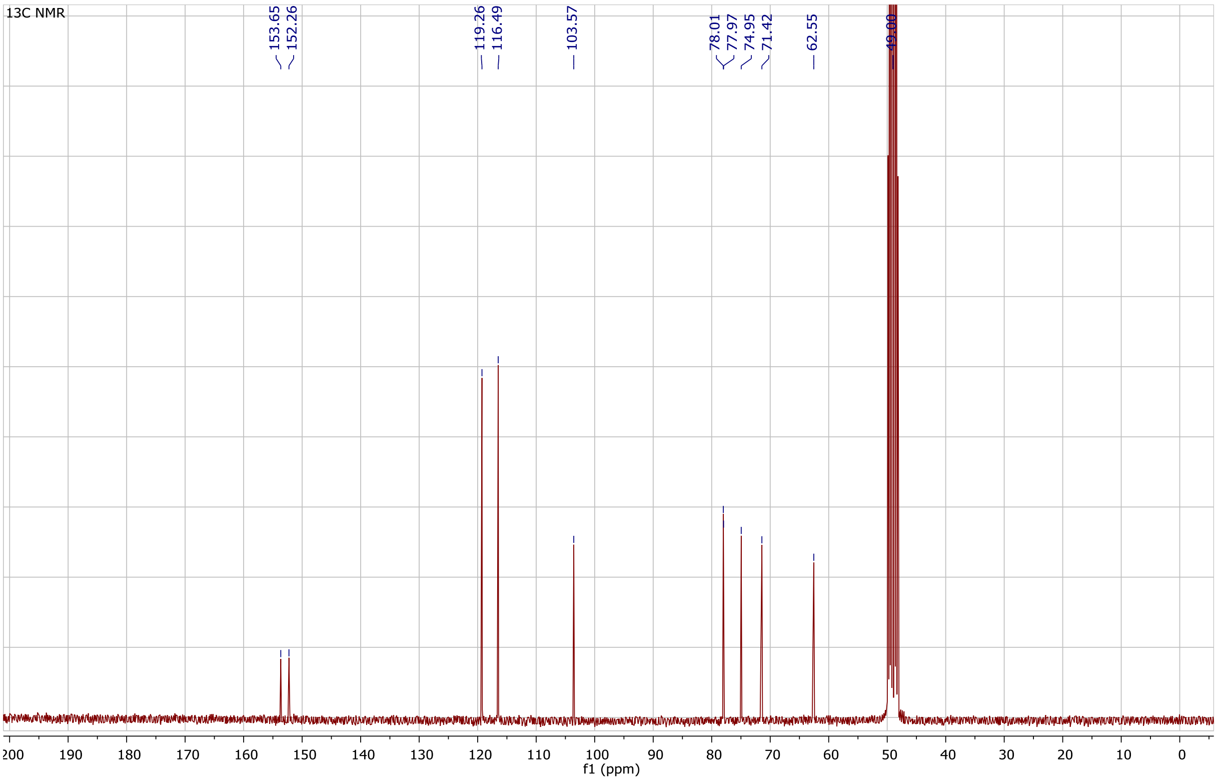

Figure S26. ^{13}C NMR spectrum (150 MHz, CD_3OD) of arbutin from *Serratulaquinquefolia*.

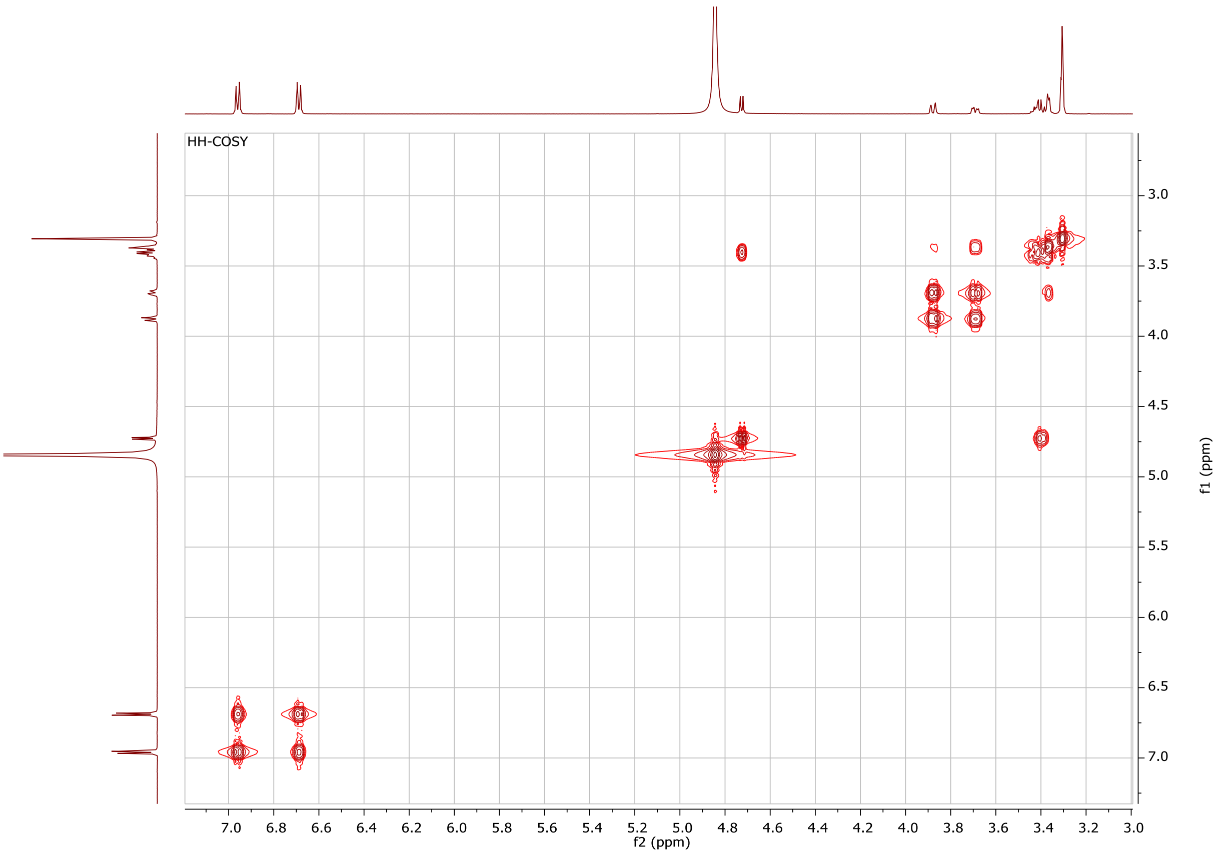

Figure S27. HH-COSY spectrum (600 MHz, CD_3OD) of arbutin from *Serratulaquinquefolia*.

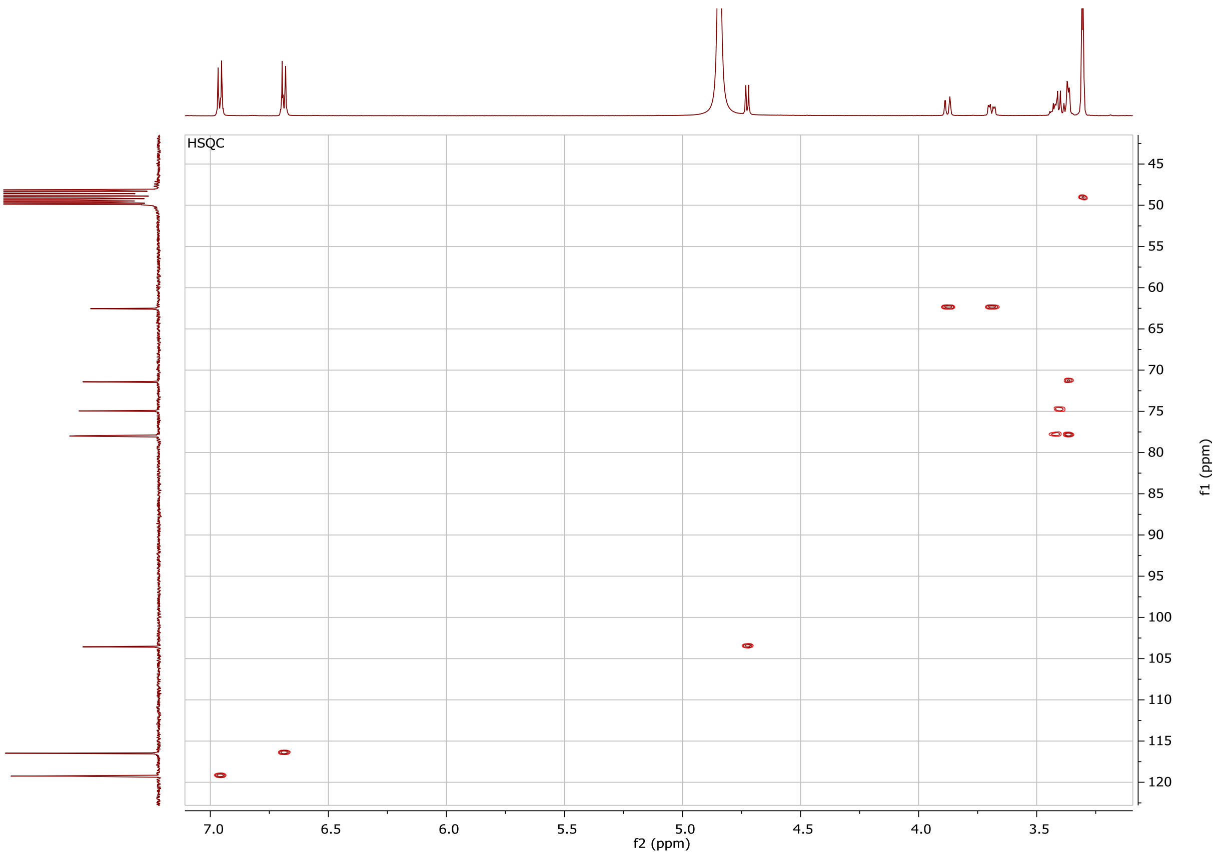

Figure S28. HSQC spectrum (600/150 MHz, CD_3OD) of arbutin from *Serratula quinquefolia*.

Table S1. NMR data (CD_3OD) of arbutin isolated from *Serratula quinquefolia.*

carbon	^{13}C<br>δ_C	^{1}H<br>δ_H (Hz)
1		-
2	118.00	6.96 d (8.9)
3	115.20	6.69 d (8.9)
4		-
5	115.20	6.69 d (8.9)
6	118.00	6.96 d (8.9)
1’	102.26	4.73 d (7.3)
2’	73.57	3.40 dd (9.0, 7.3)
3’	76.63	3.43 t (9.0)
4’	70.09	3.53 m
5’	76.63	3.53 m
6’	61.18	3.88 dd (12.0, 1.3), 3.69 dd (12.0, 5.4)
